# Single-cell RNA-seq analysis reveals ploidy-dependent and cell-specific transcriptome changes in *Arabidopsis* female gametophytes

**DOI:** 10.1186/s13059-020-02094-0

**Published:** 2020-07-22

**Authors:** Qingxin Song, Atsumi Ando, Ning Jiang, Yoko Ikeda, Z. Jeffrey Chen

**Affiliations:** 1 Department of Molecular Biosciences, The University of Texas at Austin, 1 University Station A5000, Austin, TX 78712 USA; 2 State Key Laboratory of Crop Genetics and Germplasm Enhancement, Nanjing Agricultural University, Nanjing, 210095 China; 3 Department of Biomedical Engineering, The University of Texas at Austin, 1 University Station C0800, Austin, TX 78712 USA; 4 Institute of Plant Science and Resources, Okayama University, Chuo 2-20-1, Kurashiki, Okayama 710-0046 Japan

**Keywords:** Polyploidy, Single-cell RNA-seq, Transcriptome, Gametogenesis, Reproduction

## Abstract

**Background:**

Polyploidy provides new genetic material that facilitates evolutionary novelty, species adaptation, and crop domestication. Polyploidy often leads to an increase in cell or organism size, which may affect transcript abundance or transcriptome size, but the relationship between polyploidy and transcriptome changes remains poorly understood. Plant cells often undergo endoreduplication, confounding the polyploid effect.

**Results:**

To mitigate these effects, we select female gametic cells that are developmentally stable and void of endoreduplication. Using single-cell RNA sequencing (scRNA-seq) in *Arabidopsis thaliana* tetraploid lines and isogenic diploids, we show that transcriptome abundance doubles in the egg cell and increases approximately 1.6-fold in the central cell, consistent with cell size changes. In the central cell of tetraploid plants, *DEMETER* (*DME*) is upregulated, which can activate *PRC2* family members *FIS2* and *MEA*, and may suppress the expression of other genes. Upregulation of cell size regulators in tetraploids, including *TOR* and *OSR2*, may increase the size of reproductive cells. In diploids, the order of transcriptome abundance is central cell, synergid cell, and egg cell, consistent with their cell size variation. Remarkably, we uncover new sets of female gametophytic cell-specific transcripts with predicted biological roles; the most abundant transcripts encode families of cysteine-rich peptides, implying roles in cell-cell recognition during double fertilization.

**Conclusions:**

Transcriptome in single cells doubles in tetraploid plants compared to diploid, while the degree of change and relationship to the cell size depends on cell types. These scRNA-seq resources are free of cross-contamination and are uniquely valuable for advancing plant hybridization, reproductive biology, and polyploid genomics.

## Introduction

Polyploidy or whole-genome duplication (WGD) is a widespread phenomenon that has dominated the genome evolution of many animals and all flowering plants [1–6]. Moreover, polyploid cells can form through endoreduplication during development in otherwise diploid organisms including humans [7]. The common occurrence of polyploids suggests an advantage of having additional genetic materials for evolution, adaptation, and domestication [1, 3, 5, 8, 9]. For example, yeast polyploids can obtain rapid adaptation through higher rates of beneficial mutations [10]. *Arabidopsis* autotetraploids have enhanced salinity tolerance, which is associated with elevated potassium and reduced sodium levels [11]. In *Arabidopsis* allotetraploids and *Arabidopsis thaliana* hybrids, epigenetic changes induce altered circadian rhythms, which increases photosynthesis and starch metabolism [12] and gates the timing of stress responses [13] and ethylene production [14], leading to increased growth traits such as biomass heterosis [15].

Polyploidy often leads to cell size increase as observed in yeast and *Arabidopsis* [16, 17]. However, results from gene expression studies on yeast and plant autopolyploids are inconsistent [17–20]. In yeast, ploidy variation alters a dozen of genes that regulate cell cycles and cell surface [17], while the number of genes whose expression is altered by tetraploidy varies from nine to several hundreds among different *A. thaliana* ecotypes [19, 20]. In *Glycine* species using genomic DNA normalization, the tetraploid has a 1.4-fold transcriptome abundance relative to its diploid and exhibits dosage effects on the majority of expressed genes [18, 21]. A recent study using sorted endoreduplicated nuclei in tomato fruits of diploid plants has shown a genome-wide proportional shift of gene expression depending on ploidy levels [22]. These different results may suggest that polyploid effects on gene expression vary from one genotype to another or one organism to another. Alternatively, technological limitations such as RNA-seq and microarray assays often examine relative gene expression levels and may not measure the absolute transcript abundance per gene per cell [21, 23, 24].

Single-cell RNA sequencing (scRNA-seq) analysis provides an effective alternative to study the polyploid effects on absolute levels of gene expression changes because it allows quantifying absolute transcript numbers of individual genes per cell for all genes in the genome [25, 26]. The scRNA-seq approach has been extensively used to map transcriptome dynamics from human embryos [27] to tumor evolution [28]. However, the progress on plant single-cell genomics is limited [29, 30], and transcriptome changes in polyploid plants at the single-cell level are unknown [31]. In this study, we have employed scRNA-seq technique to map absolute transcript dynamics in female gametophytic cells of *A. thaliana* diploid and isogenic autotetraploid plants, whose ploidy levels have been validated in other studies [16, 32]. The scRNA-seq results have shown ploidy-dependent and cell type-specific effects on transcriptome changes and provided unique gene expression features and valuable resources that are free of cross-contamination in the egg, central, and synergid cells during female gametophytic development.

## Results

### Experimental validation for single-cell analysis in female gametic cells

**Fig. 1:**
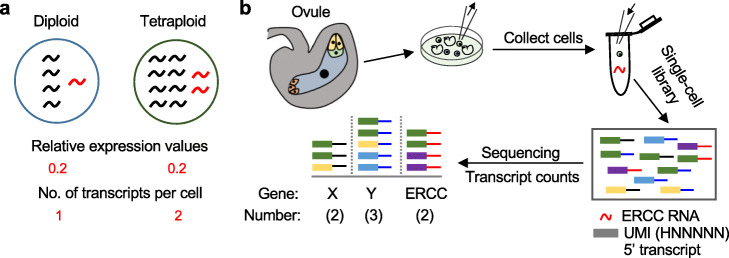
Schematic diagram of scRNA-seq analysis in diploid and tetraploid plants. **a** Relative expression level per transcriptome and absolute transcript number per cell for a gene (red) in a diploid and a tetraploid cell. **b** Pipeline of scRNA-seq analysis. Each color indicates one type of unique molecular identifiers (UMIs). ERCC, External RNA Controls Consortium. The absolute transcript level per gene is determined by the number of distinct UMIs aligned to each transcript, excluding PCR duplets, which is 2 for gene X, 3 for gene Y, and 2 for one ERCC RNA

A tetraploid cell has twice the amount of DNA relative to a diploid cell, but transcriptome studies have found a small number of genes showing expression changes between tetraploids and diploids in *Arabidopsis* [19, 20]. This is likely caused by measuring relative gene expression levels that cannot accurately measure transcriptome abundance between polyploid and diploid cells [21]. For example, if the total RNA amount doubles in the tetraploid cell relative to the diploid, the absolute number of gene transcripts would exhibit averagely twofold increase in the tetraploid relative to the diploid (Fig. 1a), whereas the relative expression level of the gene per transcriptome would be identical between diploid and tetraploid cells. In addition, some normalization methods such as spike-in RNA or genomic DNA [18, 23, 33] measure the expression level of each gene to the control (such as spike-in RNA or genomic DNA/ploidy), but not the absolute transcript numbers per gene per cell (Additional file 1: Fig. S1).

To solve this problem, we employed the scRNA-seq approach to quantify the genome-wide changes in absolute transcript levels per gene per cell in diploid and tetraploid plants (Fig. 1b). The choice of cell types is critical for the scRNA analysis. Many plant cell types often undergo endoreduplication [22], which can confound the polyploid (WGD) effect. After evaluating suitable cell types, we selected reproductive (female gametophytic) cells for the study to minimize confounding variables, and these cells are relatively uniform in a given stage. Flowering plants have three distinct female gametic cells, namely, egg, central, and synergid cells. Each egg or synergid cell is a haploid, whereas the central cell is a diploid (2 copies of the maternal genome). We generated two cell-specific lines that each expresses green fluorescent protein (GFP) in the diploid or isogenic tetraploid *A. thaliana* (Col-0) using the same construct as previously reported [34, 35]. Specifically, pDD45:nGFP and pSUP16:nGFP are expressed in the egg and central cell nuclei, respectively [34]. The synergid-cell *GFP* line was available only in the diploid plants and used for diploid comparison but excluded from the diploid-tetraploid analysis.

**Fig. 2:**
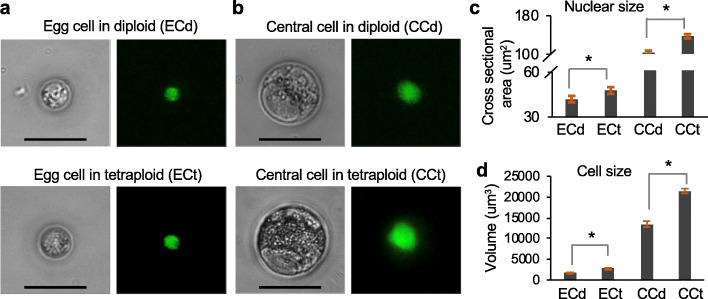
Egg and central cell size changes in diploid and tetraploid plants. **a**, **b** Representative images of egg cell (EC) (**a**) and central cell (CC) (**b**) isolated from the diploid (upper panel) and tetraploid (lower panel) *A. thaliana* (Col-0). Scale bar = 30 μm. The GFP-labeled nucleus is shown in green. Nuclear (**c**) and cell (**d**) size estimates of EC and CC with different ploidy levels using 5 cells for each cell type as in (**a)** and (**b**)

Using the nGFP marker, we manually isolated each egg cell from an ovule of flowers (stage 12) under an inverted dissecting microscope (the “Methods” section). We named the egg cell in the diploid (ECd) and tetraploid (ECt) plants (Fig. 2a). Similarly, the central cells (CCs) from the diploid and tetraploid plants are designated CCd and CCt, respectively (Fig. 2b). The egg and central cells in the tetraploid plants were ~ 1.6-fold (by volume) larger than the corresponding cells in their corresponding diploids (Fig. 2c, d), which is consistent with the cell size increase in yeast polyploids [17] and in stomatal cells of *A. thaliana* ploidy series [16]. Note that CCs are noticeably larger than the ECs in the same ovule probably because each CC is a diploid (two copies of the maternal genome) in a diploid plant or tetraploid in a tetraploid plant, while each EC is a haploid and diploid in the diploid and tetraploid plants, respectively.

For egg cell and central cell in the diploid and tetraploid plants, scRNA-seq libraries were constructed using eight cells (eight biological replicates) for each cell type as previously described [26, 36] (the “Methods” section). In addition, synergid cells in the diploid plants with four biological replicates were used for scRNA-seq library construction and analysis. An equal amount of External RNA Controls Consortium (ERCC) RNA molecules was added to each cell (Fig. 1b). During reverse transcription, each cDNA molecule was ligated with a unique molecular identifier (UMI) containing 6-bp random sequence (HNNNNN) at the 5′ end, which could be used to correct PCR-induced amplification bias, while the ligation efficiency was estimated using the external RNA controls. The raw transcript number per gene was calculated as the number of distinct UMIs aligned to the gene model, excluding duplicate counts.

Another quality control showed that the majority of sequencing reads were aligned to the transcription start sites of both endogenous genes and ERCC RNAs, indicating good preservation of mRNA integrity during RNA manipulation (Additional file 1: Fig. S2). For the expression of ERCC RNAs, the dependence of the squared coefficients of variation (CV^2^) on the molecule counts fits the Poisson distribution, which is consistent with previous single-cell RNA-seq studies [25, 37] (Additional file 1: Fig. S3). Moreover, patterns of the female gamete specifically expressed genes (6 in egg cell and 12 in central cell) from the published datasets [38–41] were reproduced in our scRNA-seq datasets (Additional file 1: Fig. S4), indicating a good reproducibility of cell-specific transcriptomes.

By counting the raw transcript number of observed UMIs, we detected ~ 25,000 transcripts per egg cell and ~ 230,000 transcripts per central cell (Additional file 2: Table S1). Based on the abundance of all ERCC spike-in controls, we found that the capture efficiency of mRNA in scRNA-seq libraries varied from ~ 2 to ~ 7%. To eliminate the effect of capture efficiency variation and more importantly to compare expression abundance among different cell types, we normalized average transcript abundance of each gene per egg or central cell using the capture efficiency (the “Methods” section). As expected, the dependence of observed molecule counts (Mol_obs_) on expected molecule counts (Mol_exp_) has fitted a Poisson generalized linear model [log_2_ (Mol_obs_) = *β*_0_ + *β*_1_log_2_ (Mol_exp_)] [42] (Additional file 1: Fig. S5). Thus, the capture efficiency and cell-to-cell technical noise were estimated using Poisson generalized linear regression of all ERCC spike-in controls for each cell (the “Methods” section). For example, the above raw numbers were normalized as average transcript abundance of ~ 449,836 per egg cell and ~ 6,924,840 per central cell. These normalized values were used for further analysis.

**Fig. 3:**
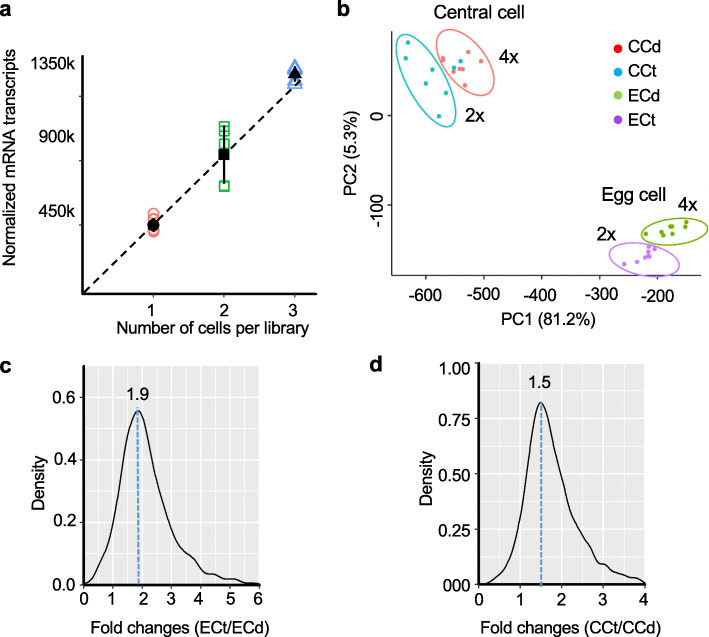
scRNA-seq analysis in egg and central cells in diploid and tetraploid plants. **a** A linear relationship between the total number (k = thousand) of normalized transcripts and the number of cells per library. Black dots indicate mean values with standard deviations of the total number of normalized mRNA transcripts estimated from 8 replicates for 1 cell and 5 replicates each for 2 and 3 cells. **b** Principal component analysis (PCA) of scRNA-seq data. ECd and ECt: egg cells in diploid and tetraploid plants, respectively; CCd and CCt: central cells in diploid and tetraploid plants, respectively. **c**, **d** Kernel density estimates of expression fold changes in the egg cells of tetraploid (ECt) and diploid (ECd) plants (**c**) and in the central cells of tetraploid (CCt) and diploid (CCd) plants (**d**)

To confirm the reproducibility, we made additional RNA-seq libraries using an artificial mix of two or three egg cells in each library, each with five biological replicates. As expected, normalized numbers of total mRNA transcripts displayed a linear relationship with cell numbers per library (from ~ 449,836 per one egg cell to ~ 1,507,362 per three cells), in spite of some variation among replicates in the two-cell sample (Fig. 3a). The correlation between the expression levels and cell numbers was further analyzed by pairwise comparison of mRNA transcript number levels among the libraries containing different numbers of cells (Additional file 1: Fig. S6a, b). Linear regression analysis showed two and three cells per library had a 1.93-fold and 3.25-fold increase of transcript numbers, respectively, compared with one cell per library; highly expressed genes showed a better correlation between transcript abundance and cell numbers than poorly expressed genes. Despite relatively low power for single-cell analysis to detect genes that are expressed at low levels, this result validated a suitability of using scRNA-seq analysis for testing transcriptome changes between different cell types of diploid and tetraploid plants.

### Transcriptome increase in the egg and central cells in tetraploid plants

Pearson’s correlation coefficients of scRNA-seq results among eight biological replicates were > 0.9 for egg cells and > 0.8 for central cells (Additional file 1: Fig. S6c), indicating low transcript number variation within the same type of female gametophytic cells. Principal component analysis (PCA) further separated the expression groups of all transcripts between cell types (egg and central cells) and between ploidy samples (diploid and tetraploid plants) (Fig. 3b). These results provided another valuable assessment of the quality and reproducibility of our scRNA-seq datasets (Additional file 2: Table S1).

For egg cells, normalized mRNA transcripts per cell were ~ 449,836 in the diploid plant (Additional file 1: Fig. S7a, b), which were doubled (~ 909,969) in the tetraploid plant (*P* = 0.0001, Monte Carlo simulation, *N* = 10,000), indicating that genome duplication has a doubling effect on the transcriptome abundance in the egg cell. Consistent with the doubling effect, the peak of fold changes in the kernel density estimation was 1.9 between ECt and ECd (Fig. 3c). However, the central cell of the tetraploid plant showed ~ 1.6-fold increase of the normalized transcript abundance (11,156,304) compared with that (6,924,840) in the diploid plant (*P* = 0.0002, Monte Carlo simulation, *N* = 10,000) (Additional file 1: Fig. S7c, d); the peak of fold changes was ~ 1.5 (Fig. 3d). At the genome-wide level, 57% and 58% of the expressed genes (> 3,000) showed larger than 1.5-fold increase after genome duplication in egg and central cells, respectively (Additional file 2: Table S1). The genes with higher expression levels tended to show more ploidy-dependent expression increase than the genes with lower expression levels (Additional file 1: Fig. S7c, d). These results suggest that genome doubling in tetraploid plants can increase overall transcript and gene expression levels, but their fold increases depend on cell types.

**Fig. 4:**
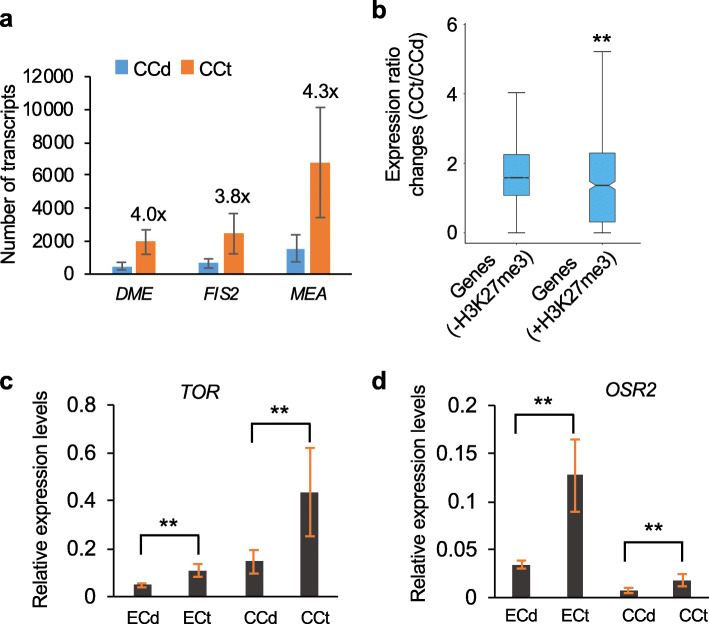
Expression validation of *PRC2* genes and *TOR* and *OSR2* in diploid and tetraploid plants. **a** qRT-PCR analysis showing increased expression levels of *DME* and *PRC2* genes (*FIS2* and *MEA*) in the central cells of tetraploid relative to diploid plants. **b** Relative expression ratio changes of the genes associated with (+) H3K27me3 (H3K27me3 target genes) or without (−) H3K27me3 (H3K27me3 non-target genes) in the central cell between tetraploid (CCt) and diploid (CCd) plants. The ratios were calculated using average expression values from eight biological replicates. The genes associated with H3K27me3 had significantly lower expression ratio changes than the genes without H3K27me3 (*P* < 1e^−7^, Wilcoxon rank-sum test). **c**, **d** qRT-PCR analysis showing increased expression levels of *TOR* (**c**) and *OSR2* (**d**) in egg and central cells of diploid and tetraploid plants. *ERCC_171* was used as an internal control, which was equally added for each cell before reverse transcription. Double asterisks indicate a statistical significance level of *P* < 0.01 (Student’s *t* test)

Although the central cell has more transcripts than the egg cell, the fold increase of transcriptome changes in the tetraploids is smaller in the former (1.6-fold) than in the latter (2-fold). This could be related to genome-wide demethylation and de-repression of chromatin and/or transcriptional repressors in the central cell. Coincidently, the central cell-specific DNA hypomethylation factor, *DEMETER* (*DME*) [43], was expressed 4-fold higher in the tetraploid plant than in the diploid (Fig. 4a). DME activates Polycomb Repressive Complex 2 (*PRC2*) genes such as *fertilization-independent seed 2* (*FIS2*) [44] and *MEDEA* (*MEA*) [45] in the central cell [46]. As a result, expression levels of *FIS2* and *MEA* were increased from 3.8- to 4.3-fold in the tetraploid plant than in the diploid (Fig. 4a). Activation of *PRC2* genes can suppress the transcription of many other genes through the induction of histone H3K27 trimethylation (H3K27me3) [47, 48]. Indeed, H3K27me3 target genes identified in a previous study using the endosperm [49] showed significantly lower fold changes than the genes without H3K27me3 in the central cell of tetraploid plants (*P* < 1e^−7^, Wilcoxon rank-sum test) (Fig. 4b). These results suggest that activation of *PRC2* genes may contribute to H3K27me3 increase and repression of overall expression levels in the central cell of tetraploid plants. Moreover, increased expression levels of *PRC2* genes in the central cell prior to fertilization could help bypass (endosperm) barriers of interspecific hybridization in allopolyploids as previously predicted [50, 51]. Notably, these genes (*DME*, *FIS2*, and *MEA*) are expressed specifically in the central cell and later in the endosperm but not in the egg cell [38] (Additional file 2: Table S1).

Among the upregulated genes by genome doubling, expression changes of 1,344 and 1,978 genes had larger than a 2-fold increase (*P* < 0.05, one-way ANOVA) in egg and central cells, respectively (Additional file 3: Table S2). Gene Ontology (GO) analysis showed enrichment of these genes in transcription- and translation-related processes, including translational initiation, mRNA processing, and protein folding (Additional file 1: Fig. S8). The larger size of the egg and central cells in the tetraploid than in the diploid led us to examine whether regulators of cell expansion were also upregulated after genome duplication. *Arabidopsis* has several key factors involved in cell expansion and size, including ARGOS-LIKE (ARL) [52], TARGET OF RAPAMYCIN (TOR) [53], THESEUS1 (THE1) [54], and ORGAN SIZE RELATED 2 (OSR2) [55]. *ARL* and *THE1* showed very low or no expression in the egg and central cells of diploid plants (Additional file 2: Table S1). Interestingly, expression levels of *OSR2* and *TOR* in the egg and central cells were increased twofold or more in the tetraploid plants relative to the diploid ones (Additional file 2: Table S1); the result was further validated by qRT-PCR (Fig. 4c, d). These data suggest that the cell size regulators may also contribute to cell size increase in response to a ploidy increase.

Consistent with the increased cell size by ploidy, the central cell (diploid) is larger than the egg cell (haploid) from the same diploid plant, albeit of their different cell types (Fig. 2). The central cell had 6,924,840 normalized mRNA transcripts, 15-fold higher than the egg cell in the same diploid plant (Additional file 1: Fig. S7a, c). In addition, the synergid cell in the diploid plant had ~ 66,773 raw transcripts or ~ 2.9 million normalized mRNA transcripts. This suggests that the synergid cell contains more transcripts than the egg cell but less than the central cell, which is consistent with the published observation that the synergid cell is larger than the egg cell but smaller than the central cell [56]. These results suggest a positive correlation between RNA content and cell size [57]. The disproportionally larger than twofold increase in the transcriptome abundance among cell types probably reflects different activities in cell types; central cells are more metabolically active than the egg cells [58].

### Cell-specific gene expression in egg, central, and synergid cells in diploid plants

**Fig. 5:**
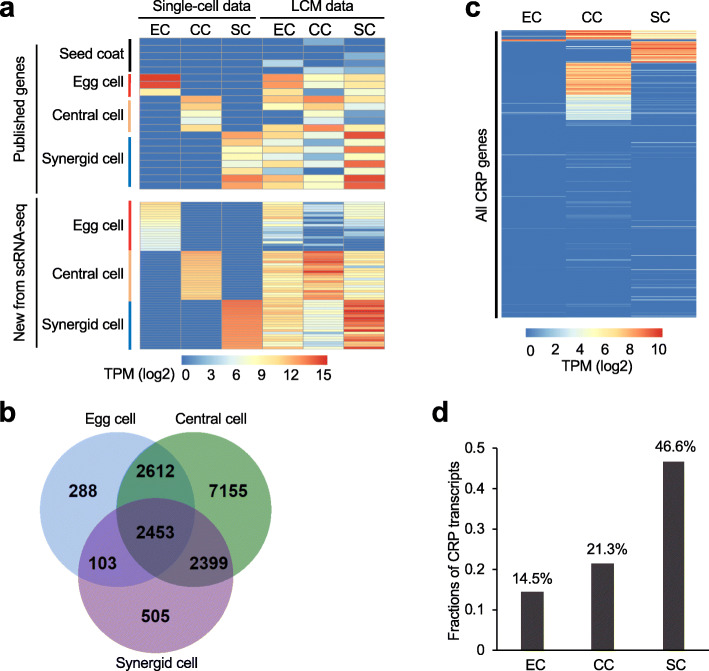
Gene expression patterns in the female gametes revealed by scRNA-seq analysis. **a** Clustering analysis of female gamete-expressed genes in LCM datasets [64, 65] and in scRNA-seq data (this study) (upper panel) and new sets of female gamete-expressed genes (lower panel) identified by scRNA-seq analysis. Gene expression levels in scRNA-seq were mean transcript per million (TPM) values of the genes in all cells for each cell type. EC, egg cell; CC, central cell; SC, synergid cell. **b** Venn diagram showing the numbers of the genes that are expressed in three female gametophytic cells. An expressed gene is defined as its expression in one or more cells examined. **c** Expression clustering of all CRP (cysteine-rich peptides) genes in EC, CC, and SC. **d** Fractions of CPR transcripts out of total mRNA transcripts in EC, CC, and SC

Previous studies have generated transcriptome data using micro-dissected female gametophytes or sporocyteless mutant in *Arabidopsis* [59], wheat [60], rice [61], and maize [62]. In *Arabidopsis*, laser capture microdissection (LCM) combined with microarray or RNA-seq was commonly used to study gene expression changes in female gametophytic cells [63–65], which could result in datasets with mRNA cross-contamination among different cell types [66]. The quality of our scRNA-seq data was tested in the egg cell, central cell, and synergid cell (Fig. 5a). For comparative analysis, both scRNA-seq (this study) and published LCM-RNA-seq [64, 65] datasets were normalized by transcripts per million (TPM) (the “Methods” section). As expected, a subset of known gamete-specifically expressed genes (AT2G21740, AT1G74480, and AT2G21750 in EC; AT5G38330, AT3G10890, AT4G25530, AT5G04560, and AT3G04540 in CC; and AT1G47470, AT5G43510, AT4G18770, AT5G42955, AT4G07515, AT1G52970, AT2G21655, and AT5G12380 in SC) as reported [34, 41], also exhibited cell-specific expression patterns in our scRNA-seq datasets (Fig. 5a, upper panel). However, expression patterns of these genes were mixed among cell types in the LCM-RNA-seq datasets (Fig. 5a, upper right panel). The scRNA-seq datatsets were largely contamination free, whereas LCM datasets included some seed coat genes (AT1G61720, AT5G48100, AT5G35550, AT4G09960, and AT5G17220) with low expression levels in female gametes.

In scRNA-seq datasets, we identified 5,456, 14,619, and 5,460 genes that were expressed (read count > 0 in any of the libraries) in the egg, central, and synergid cells, respectively (Fig. 5b). A total of 12,738 genes (39% of all expressed genes) showed significantly different expression patterns in at least two of the three (egg, central, and synergid) cell types (*P* < 0.05, one-way ANOVA), indicating enormous expression variation among female gametes. Compared to the egg and synergid cells, the central cell had 2.7-fold more expressed genes, suggesting higher metabolic and biological activities in the central cell (Fig. 5b). Among those genes expressed in cell types, 288, 505, and 7,155 were exclusively expressed in the egg cell, synergid cell, and central cell, respectively (Fig. 5b), including top 20 genes with the highest expression values that are uniquely expressed in each cell type (Fig. 5a, lower left panel). The genes with expression patterns shared among cell types include 2,453 in all three cell types, 2,612 in EC and CC, 103 in EC and SC, and 2,399 in CC and SC (Fig. 5b). GO enrichment analysis of the female gamete-expressed genes indicated that some biological processes are shared among three female cell types, while others are specific to each cell type: embryo development in the egg cell, photosynthesis and response to cytokinins in the central cell, and pollen germination and small GTPase-mediated signal transduction in the synergid cell (Additional file 1: Fig. S9). These results are consistent with respective biological roles in three cell types: embryo development in the egg cell, photosynthesis and energy metabolism in the central cell (progenitor of the endosperm), and assisting pollen tube growth and fertilization in the synergid cell.

**Fig. 6:**
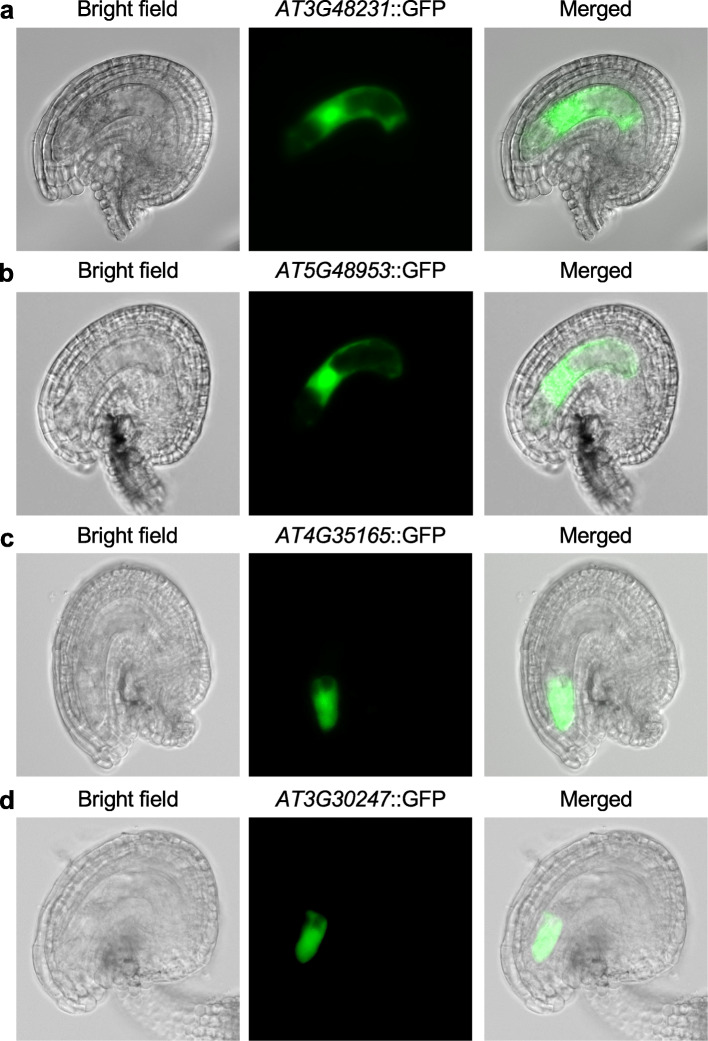
Validation of expression patterns of *CRP* genes in promoter::GFP transgenic lines. Expression of AT3G48231 (**a**) and AT5G48953 (**b**) in the central cell. AT3G48231 (low-molecular-weight cystine-rich 48): LCR48; AT5G48953*:* LCR86. Expression of AT4G35165 (**c**) and AT3G30247 (**d**) in the synergid cell. AT4G35165, a *CRP-*encoded egg cell-secreted-like protein; AT3G30247, a *CRP* encoding an ECA1 (early culture abundant 1) gametogenesis-related family protein

Remarkably, a group of gene family members encoding cysteine-rich peptides (CRPs) [67], accounted for a large amount of total normalized mRNA transcripts in the egg (15%), central (21%), and synergid (46%) cells (Fig. 5c, d). CRPs regulate plant growth and development through modulation of cell-cell communications, including the guidance of pollen tube and gamete recognition during fertilization [58, 68]. Among these CRP genes (Additional file 4: Table S3), we randomly selected four newly defined genes (two expressed in the synergid cell and two expressed in the central cell) for functional validation by expressing promoter::GFP in the transgenic lines (Fig. 6). Consistent with the scRNA-seq data, two genes (AT5G48953 and AT3G48231) were expressed in the central cell (Fig. 6a, b), according to the image evaluation methods as previously described [34], while the other two (AT4G35165 and AT3G30247) were expressed in the synergid cell (Fig. 6c, d). The data suggest roles of these cell-specifically expressed genes in female-male gametic interactions between synergid cell and pollen tube (sperm) for pollen guidance and between central cell and sperm in double fertilization for endosperm development.

## Discussion

Single-cell analysis provides new opportunities to study complex cellular systems at the individual cell resolution. It is used not only to dissect cell heterogeneity in given tissues, but also to quantify absolute expression level in each cell. In this study, we have employed scRNA-seq to examine the relationships between ploidy, cell size, and transcriptome abundance. Cell types and sizes vary from one tissue to another in plants. For example, the commonly used leaf cells are often mixed with diploid and endoreduplicated cells [16], which could confound the effects of polyploidy (WGD) and endoreduplication [22]. Although cell types are well-defined in root cells [69], they show large gene expression variation from one cell type to another from > 26-fold in the epidermal cell to > 3-fold in the quiescent center cell [25]. In our study, cell size and expression values of female gametophytic cells show less variation from one plant to another in similar stages at the same ploidy level (Fig. 2 and Additional file 1: Fig. S7). This is probably because the synchrony of gametes is critical to fertilization and embryo development. For example, cell cycle regulation is coordinated between male and female gametes for double fertilization [70]; both egg and central cells are arrested at the G1/S transition in tobacco [71].

Our data indicate that in the tetraploid plants relative to the diploids, overall transcript abundance per cell is doubled in the egg cell and increased in the central cell, which is consistent with the cell size increase in these cells. This conclusion is different from previously published results in which fewer genes are upregulated in response to ploidy increase in yeast ploidy series [17] and in *Arabidopsis* autotetraploids [19]. When cells are sorted by the nuclei of diploid and endoreduplicated cells in tomato pericarps, the transcriptome abundance is correlated with the ploidy level of the cells [22]. Cell size is positively correlated with transcriptome abundance, as observed between transcriptome abundance and cell size in yeast, plant, and mammals [21, 72]. In yeast, genes controlling cell cycles, *Cln1* and *Pcl1*, are repressed as the ploidy levels increase, leading to larger cell size [17]. In *Arabidopsis*, because these female gametic cells do not divide, the cell size increase is likely related to cell expansion [73, 74]. This cell expansion regulation involves several key factors, including OSR2 [55] and its family member ARL [52], TOR kinase [53], and receptor-like kinase THESEUS1 (THE1) [54]. TOR kinase is evolutionarily conserved and functions to mediate ribosomal biogenesis and translation that promote cell proliferation and growth including embryogenesis and life span determination [75]. Ectopic expression of *TOR* [53] or *OSR2* [55] results in larger cells than the wild type, while downregulation of *ARL* inhibits cell growth [52]. *THE1* is required for cell elongation through brassinosteroid-mediated signaling, and downregulation or mutation of *THE1* results in dwarf plants [54]. Thus, in addition to the polyploid effect, cell size regulators such as *TOR* [53] or *OSR2* [55] may also affect cell size increase in the egg and central cells. Increased expression of these genes in the egg and central cell of tetraploid plants relative to the diploids suggest their potential roles in cell size variation, although the conclusion for a causal effect should await further experimentation.

The variation of transcriptome abundance increase between the cell types is likely associated with the biological activity of each cell type. In egg cells that maintain a quiescent state, genome doubling increases the cell size and doubles the transcriptome. In the central cell, epigenetic reprogramming by the activation of *DME*, which is not expressed in the egg cell, could alter the overall impact of transcriptome changes. Interestingly, *DME* [45] is upregulated in the central cell of tetraploid plants relative to the diploids (Fig. 4a); this could activate general transcriptional repressors such as *PRC2* gene families including *FIS2* [44] and *MEA* [45] that can promote H3K27 trimethylation of many PRC2 target genes in the central cell [47, 48]. Consistently, the expression fold change of H3K27me3 target genes was significantly lower than that of H3K27me3 non-target genes in the central cell between tetraploid (CCt) and diploid (CCd) plants (Fig. 4b). As a result, the overall increase of transcript abundance is not exactly to the twofold ploidy level as observed in the egg cell. Although this prediction is based on the correlated data of gene expression, it can be tested by examining the transcriptional activity of egg and central cells in the *dme* mutant. Unfortunately, in spite of concerted and collaborative efforts, we could not isolate intact central cells in the *dme* line, and the central cells were easily broken with dissipated nuclei. It is likely that the disruption of *DME* or *PCR2* complex genes may affect the cellular morphology and make the central cells sensitive to physical manipulations.

Compared to previous studies, our scRNA-seq datasets are largely free of cross-contamination among three female gametophytic cells, which provide a unique resource for studying plant reproductive biology. Many genes with cell-specific expression patterns in each female gamete identified in this study can provide useful clues for better understanding the molecular events of cell-cell recognition during fertilization. CRPs are involved in diverse aspects of cell-cell communication during vegetative growth and plant reproduction [58, 68]. As the amino acid composition of CRPs is highly divergent, different CRPs may play specific and unique functions in the egg cell, central cell, or synergid cell during fertilization. For example, CRPs secreted from synergid cells can provide guidance for pollen tube and sperm cell release, while those from the central cell and egg cells can attract sperm to produce embryo and endosperm of a seed [58, 71], a process known as double fertilization [70, 76]. These newly identified female gamete-expressed CRPs are key signaling factors in regulating reproductive development in plants. Compared to the egg cell, the function of CRPs in the central cell and synergid cell is poorly understood. Our scRNA-seq resource should provide valuable guidelines for future research directions to elucidate the roles of egg, central, and synergid cells during double fertilization [58, 68]. Understanding the molecular mechanisms for the polyploid effects on cell and organ size and for signaling processes during double fertilization will help us develop tools to overcome hybridization barriers as well as to improve polyploid plants, many of which are important crops.

## Methods

### Isolation of egg, central, and synergid cells in *A. thaliana*

*A. thaliana* (Col-0) diploids and tetraploids were obtained by colchicine treatment and confirmed by chromosome spreads and flow cytometry [16]. The same batch of seed stocks was used for this study. Diploid and tetraploid plants were independently transformed with each of the constructs. Transgenic plants (*A. thaliana* Col-0) showing cell-specific promoter:nGFP of pDD45:nGFP (in egg cell nucleus) [34], pSUP16:nGFP (in central cell nucleus) [35], or pDD31:nGFP (in synergid cell nucleus) [35] were generated in *A. thaliana* (ecotype Col-0) diploid and isogenic tetraploids [16]. All transgenic plants were grown under the long-day condition (light/dark cycle of 16/8 h) at 22 °C. Flowers at stage 12 were tagged and emasculated, and the emasculated plants were grown for another day. The ovules were dissected from pistils and soaked in a protoplast enzyme solution (2% cellulose, 0.3% macerozyme R-10, 0.05% pectolyase, and 0.45 M mannitol) for 15 min as previously described [77]. Individual egg, central, or synergid cells labeled independently by nucleus-localized GFP were carefully isolated from different plants in various times and captured into a microtube using a microcapillary pipette that is installed in a micromanipulator (MN-151, NARISHIGE) and a micro-injector (IM-11-2, NARISHIGE) under an inverted dissecting microscope (Eclipse Ts2R, Nikon).

### Single-cell RNA-seq library construction and qRT-PCR analysis

scRNA-seq libraries were constructed using a modified protocol as previously described [26, 36]. Each single cell was placed into 4 μl lysis buffer (0.1% Triton X-100, 1 U/μl RNaseOUT Ribonuclease Inhibitor (Thermo Fisher Scientific, Waltham, MA), 4 μM oligo-dT primer (5′-GTTCAGAGTTCTACAGTCCGACGATCGTTTTTTTTTTTTTTTTTTTTTTTTTTTTT-3′), 2.5 mM dNTP, and 1 μl 1:3,000,000 ERCC Spike-In Mix 1 (Thermo Fisher Scientific, Waltham, MA) with ~ 20,775 RNA molecules). Samples were incubated at 72 °C for 3 min and immediately placed on ice. After lysis, each sample was added by 5.6 μl of RT mix, consisting of 2 μl Superscript II first-strand buffer (5×, Thermo Fisher Scientific), 0.5 μl DTT (100 mM, Thermo Fisher Scientific), 2 μl Betaine (5 M, Sigma-Aldrich, St. Louis, MO), 0.1 μl MgCl_2_ (1 M, Sigma-Aldrich), 0.25 μl TSO primer (100 μM, 5′-GUUCAGAGUUCUACAGUCCGACGAUCHNNNNNGGG-3′), 0.25 μl RNaseOUT Ribonuclease Inhibitor, and 0.5 μl SuperScript II reverse transcriptase (200 U/μl, Thermo Fisher Scientific). Reverse transcription reaction was performed by incubating samples at 42 °C for 90 min, followed by 10 cycles of reverse transcription (50 °C for 2 min and 42 °C for 2 min) and one cycle of extension (72 °C for 15 min). The cDNA was amplified by adding 15 μl PCR mix (12.5 μl KAPA HiFi HotStart ReadyMix (2×, Kapa Biosystems, Wilmington, MA), 0.25 μl IS PCR primer (10 μM, 5′-GTTCAGAGTTCTACAGTCCGACGATC-3′), and 2.25 μl water) and incubating at a thermal cycler as follows: 98 °C for 3 min; 18–20 cycles of 98 °C for 20 s, 68 °C for 15 s, and 72 °C for 6 min; and 72 °C for 5 min. PCR product was purified using AMPure XP beads (1:1 ratio; Beckman Coulter) and then used for qRT-PCR or library construction.

For qRT-PCR, 100 pg amplified DNA was used as the template, and the reaction was run on the LightCycler 96 System (Roche, Indianapolis, ID). The relative expression level was quantified using internal control *ERCC_ 171* with six biological replicates, and three technical replicates were used for each biological replicate. Primers for qRT-PCR were listed in Additional file 5: Table S4.

For library construction, 200 pg amplified DNA was tagmented using Nextera XT DNA sample preparation kit according to the manufacturer’s instructions (Illumina, San Diego, CA). The fragmented DNA was purified using AMPure XP beads (1:1 ratio; Beckman Coulter) and incubated with PvuI-HF enzyme (NEB, Ipswich, MA) for 30 min at 37 °C. After digestion by PvuI-HF, DNA was purified using AMPure XP beads (1:1 ratio; Beckman Coulter) and suspended in 18 μl water. Final amplification was performed by adding 22 μl PCR mix (20 μl KAPA HiFi HotStart ReadyMix (2×, Kapa Biosystems, Wilmington, MA), 1 μl FP PCR primer (10 μM, 5′-AATGATACGGCGACCACCGAGATCTACACGTTCAGAGTTCTACAGTCCGA-3′), and 1 μl RP PCR primer (10 μM, 5′-CAAGCAGAAGACGGCATACGAGATXXXXXXGTCTCGTGGGCTCGG-3′ (XXXXXX indicates index sequence) and incubating samples at a thermal cycler as follows: 98 °C for 3 min; 12–15 cycles of 98 °C for 20 s, 65 °C for 30 s, and 72 °C for 1 min; and 72 °C for 5 min. Final libraries were purified using AMPure XP beads (0.8:1 ratio; Beckman Coulter) and sequenced using NextSeq 500 platform (Single-end 75-bp reads).

### Read mapping of scRNA-seq datasets

We first removed low-quality reads that showed a quality score of 29 or lower in the first six bases using the NGS QC Toolkit (version 2.3) and then discarded reads without the UMI pattern HNNNNNGGG at 5′ ends. The remaining reads were mapped to the pseudogenome consisting of *Arabidopsis* genome (TAIR 10) and ERCC sequences using STAR with parameters (--outFilterMismatchNoverLmax 0.02), and non-uniquely mapped reads were discarded [78]. To account for the incomplete information of transcription start sites, the 5′ ends of all gene models were extended by 100 bases but not beyond the 3′ ends of upstream genes. Each unique UMI barcode represents one RNA transcript. Errors in the UMI sequence generated during PCR and sequencing could create additional artificial UMIs. To account for sequencing errors and remove PCR duplicates, UMI-tools were applied to improve the quantification accuracy [79]. Molecule count of each gene was calculated as the total number of distinct UMIs mapped to the corresponding gene model.

### Normalization of average transcript abundance in each scRNA-seq library

The dependence of observed molecule counts (Mol_obs_) on expected molecule counts (Mol_exp_) fitted a Poisson generalized linear model for ERCC spike-in controls (Additional file 1: Fig. S5). To account for capture efficiency variation and technical noise between different cells, we first fitted a Poisson generalized linear model (GLM) using the ERCC spike-in controls:

$$ \log 2\left(\mathrm{Mo}{\mathrm{l}}_{\mathrm{obs}}\right)={\beta}_0+{\beta}_1\log 2\left(\mathrm{Mo}{\mathrm{l}}_{\mathrm{exp}}\right) $$

where Mol_exp_ is the expected ERCC molecule counts, and Mol_obs_ is the observed raw ERCC molecule counts [42]. The slope (*β*_1_) and intercept (*β*_0_) of ERCC spike-in controls in each library were calculated using the *glm* function in R software as “glm(*y*~log2(*x*), family = ‘Poisson’)” in which *x* and *y* were the Mol_exp_ and Mol_obs_, respectively, of the full set of ERCC spike-in transcripts. Endogenous genes were expected to show a similar relationship between observed raw molecule counts and actual molecule counts as ERCC spike-in controls. Then, we transformed observed molecule counts of endogenous genes (Mol_obs_gene_) with slope (*β*_1_) and intercept (*β*_0_) of the Poisson GLM regression line to generate the normalized expression levels of genes (Mol_norm_gene_) for each cell:

$$ \log 2\left(\mathrm{Mo}{\mathrm{l}}_{\operatorname{norm}\_\mathrm{gene}}\right)=\left(\log 2\left(\mathrm{Mo}{\mathrm{l}}_{\mathrm{obs}\_\mathrm{gene}}\right)-{\beta}_0\right)/{\beta}_1 $$

### Identification of upregulated genes by genome doubling

Data in each cell was treated as a biological replicate. Normalized expression abundance of each gene with eight replicates was subjected to a one-way ANOVA test using *f_oneway* function in the SciPy software (https://www.scipy.org) to identify upregulated genes between ECt and ECd or between CCt and CCd with a statistical significance level of *P* < 0.05.

### Principal component analysis and Gene Ontology analysis

Normalized expression abundance of genes in egg cells and central cells from diploid and tetraploid plants was subject to logarithm transformation using *log2* function in R software. PCA was performed using the *prcomp* function in R software with the parameters “scale. = FALSE, center = FALSE, tol = 0”.

GO analysis was performed using the Database for Annotation, Visualization and Integrated Discovery (DAVID) v6.8 (https://david.ncifcrf.gov/home.jsp) [80]. The enriched biological process terms were extracted from the GOTERM_BP_DIRECT category with Benjamini-corrected *P* value < 0.05.

### Analysis of published LCM datasets and genes expressed in female gametes

Raw reads from LCM datasets were mapped to *Arabidopsis* genome (TAIR 10) using the HISAT2 (v2.1.0) software with default parameters [81]. Uniquely mapped reads were extracted and used to calculate transcripts levels (transcripts per million (TPM)) of each gene through the StringTie (v1.3.3) software [82].

To compare our scRNA-seq datasets with the LCM datasets, we calculated the TPM value of each gene in scRNA-seq datasets. For each gene, the total number of distinct UMIs was calculated as the transcript number. The sum of transcript numbers of all genes was divided by 1,000,000 to create a scaling factor. Transcript number of each gene is divided by the scaling factor to generate the TPM value of each gene.

### Expression validation of CRP genes

Genomic DNA from *Arabidopsis* (Col-0) was used to amplify promoter regions of *AT3G4823*, *AT5G48953*, *AT4G35165*, and *AT3G30247*. Primer pairs are listed in Additional file 5: Table S4. The amplified fragments were cloned into pBI-n1GFP vector. All constructs were individually cloned into *Agrobacterium* strain GV3101 and then transformed into diploid *Arabidopsis* (Col-0) using the standard floral dip method [83]. GFP activity within the mature female gametophyte was captured 1 day after emasculation using Zeiss Axiovert 200M fluorescence microscope.

## Supplementary information

**Additional file 1:** Supplementary Figures S1–S9.

**Additional file 2:Table S1.** Expression values (raw reads and normalized abundance) of each gene in each cell.

**Additional file 3:Table S2.** List of differentially expressed genes after genome duplication at a statistical significance level (*P* < 0.05, one-way ANOVA) using normalized expression values.

**Additional file 4:Table S3.** Normalized expression values of each CRP gene in each cell.

**Additional file 5:Table S4.** Primer pairs used for qRT-PCR and GFP validation.

**Additional file 6:** Review history.

## Data Availability

scRNA-seq data were deposited in the NCBI Nucleotide and Sequence Read Archive (SRA) under Accession SRP160651 [84]. All codes used for statistical analysis are available upon request or at GitHub [85].
