# Supplementary material for: Single-cell RNA-seq analysis reveals ploidy-dependent and cell-specific transcriptome changes in Arabidopsis female gametophytes
Source: Genome Biol. 2020 Jul 22;21:178. doi: 10.1186/s13059-020-02094-0 (PMC7375004; doi:10.1186/s13059-020-02094-0)
Supplement: Supplementary file 1 — Additional file 1: Supplementary Figures S1–S9. [file 13059_2020_2094_MOESM1_ESM.pdf]

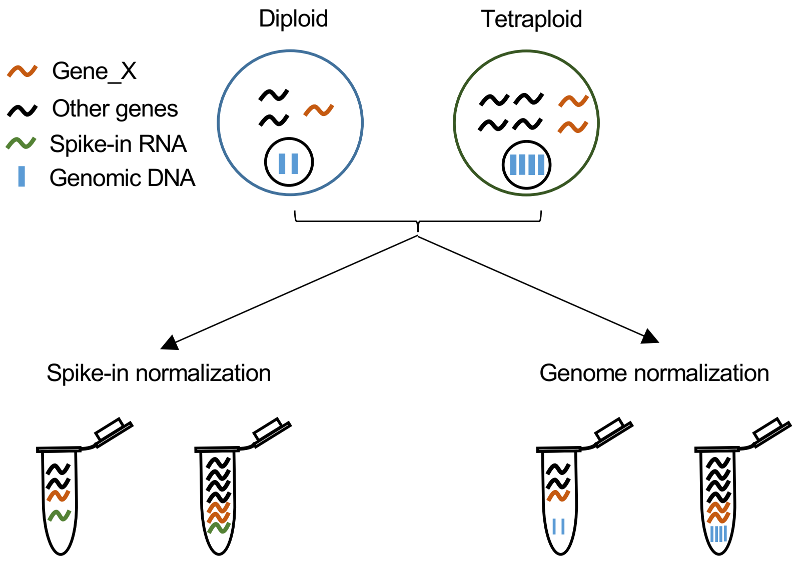

Method	Gene_X / Spike-in RNA	Gene_X / Spike-in RNA	Gene_X / genomic DNA	Gene_X / genomic DNA
Relative expression per cell	1	2	0.5	0.5

Additional file 1: Figure S1. Relative gene expression levels per cell may vary and depend on different normalization methods. In spike-in normalization, the equal proportion of external RNAs to cell number is added to each sample. Expression levels of individual genes are normalized by the external RNAs. In genomic DNA normalization, genomic DNA is co-extracted with RNA from each sample. The expression level of each gene is normalized by the amount of genomic DNA. The model were modified from Coate and Doyle's paper (Coate and Doyle, 2015, Chromosoma).

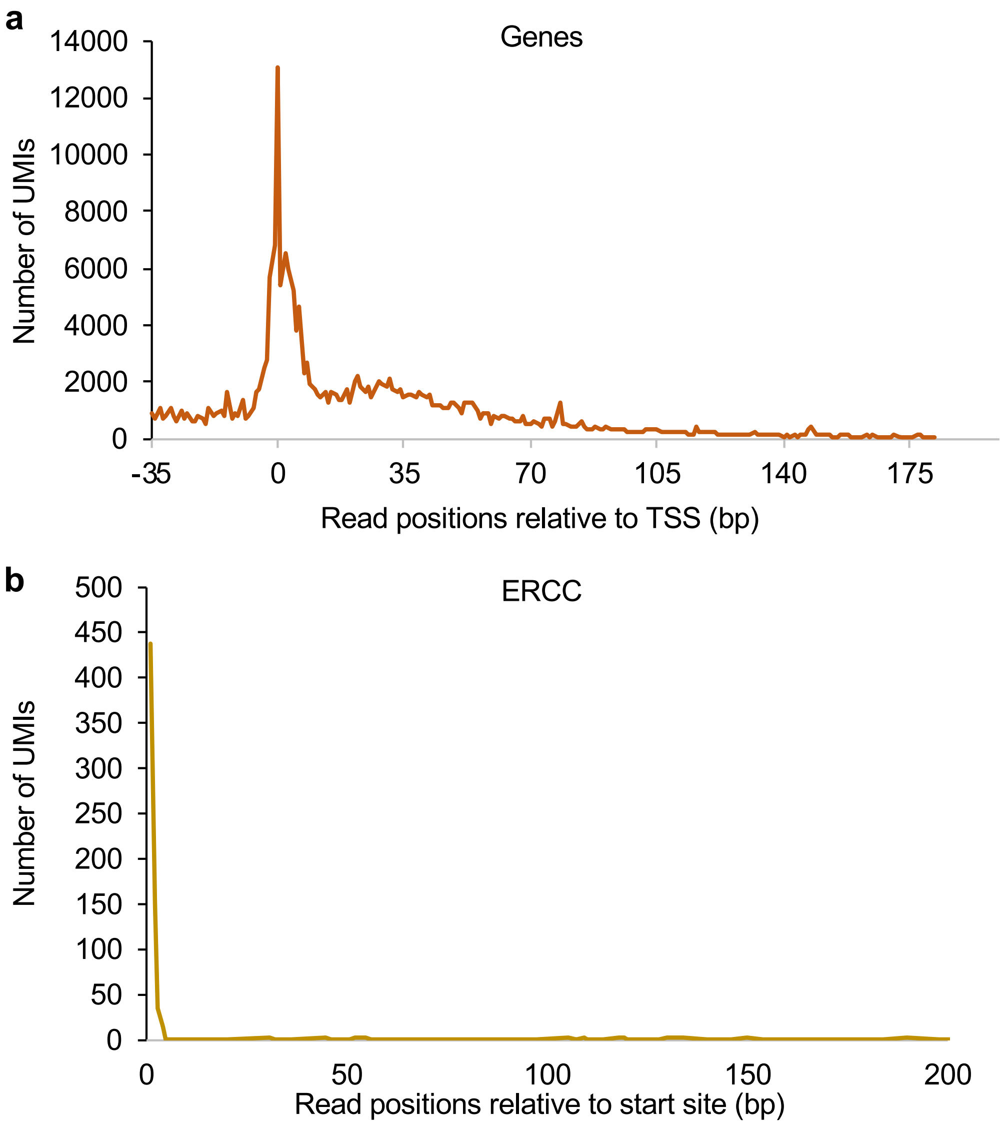

Additional file 1: Figure S2. scRNA-seq read distribution. **a** Distribution of scRNA-seq reads around the transcription start sites (TSSs) of endogenous genes. **b** Distribution of reads around the ERCC control RNA transcripts.

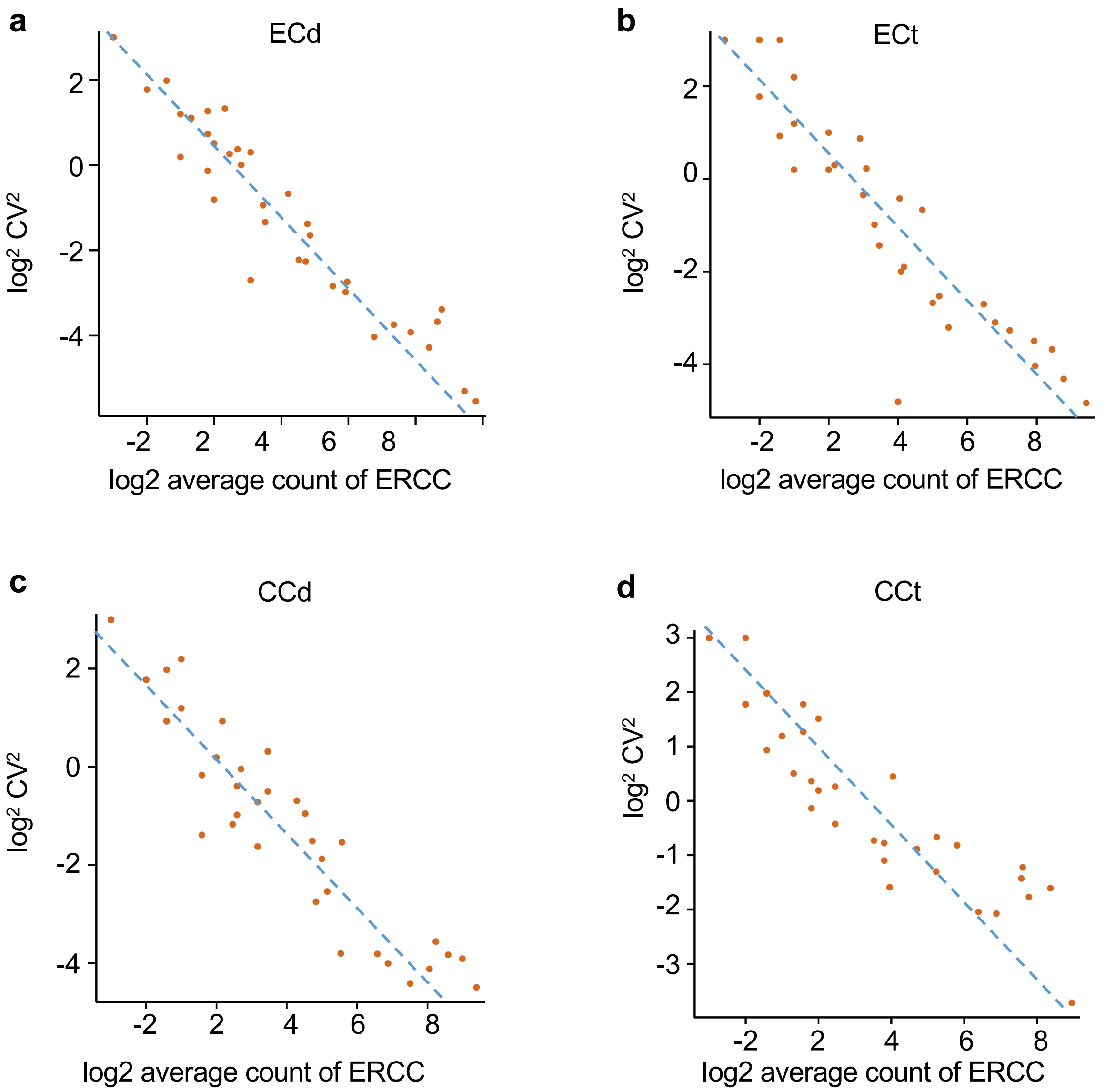

Additional file 1: Figure S3. Estimates of transcript numbers using Poisson distribution. **a-d** Squared coefficients of variation (CV^2) are plotted against the means of read counts for each ERCC RNA in ECd (**a**), ECt (**b**), CCd (**c**) and CCt (**d**). Blue line indicates CV^2 for a hypothetical Poissonian distribution.

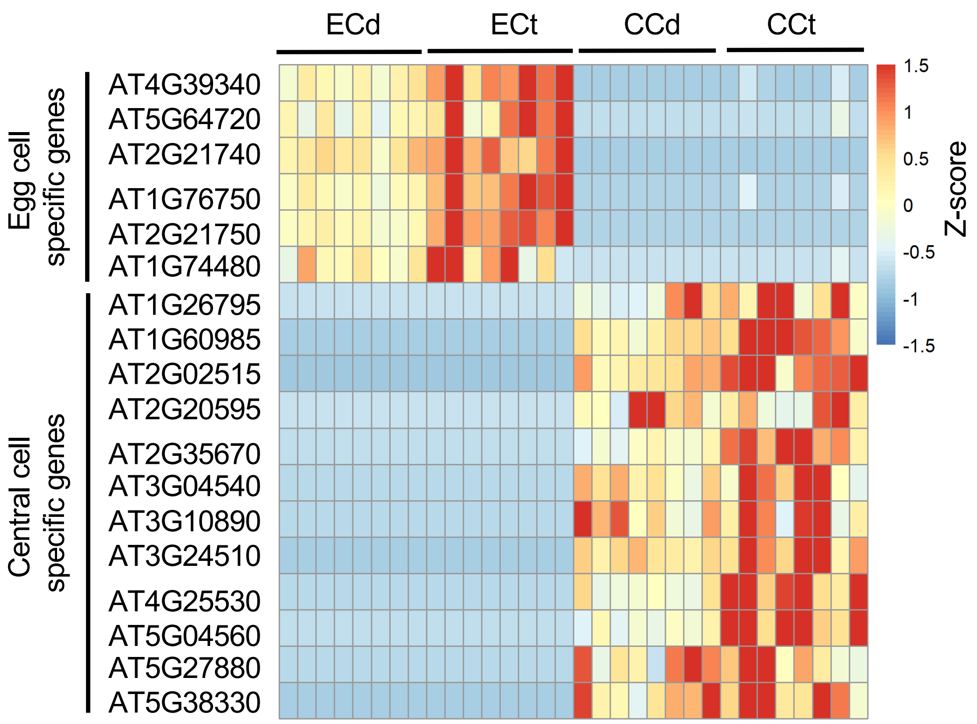

Additional file 1: Figure S4. Clustering analysis of published data and scRNA-seq data in egg and central cells. Absolute expression levels of published female gamete-specific genes in ECd, ECt, CCd and CCt using six egg cell-specific genes and 12 central cell-specific genes.

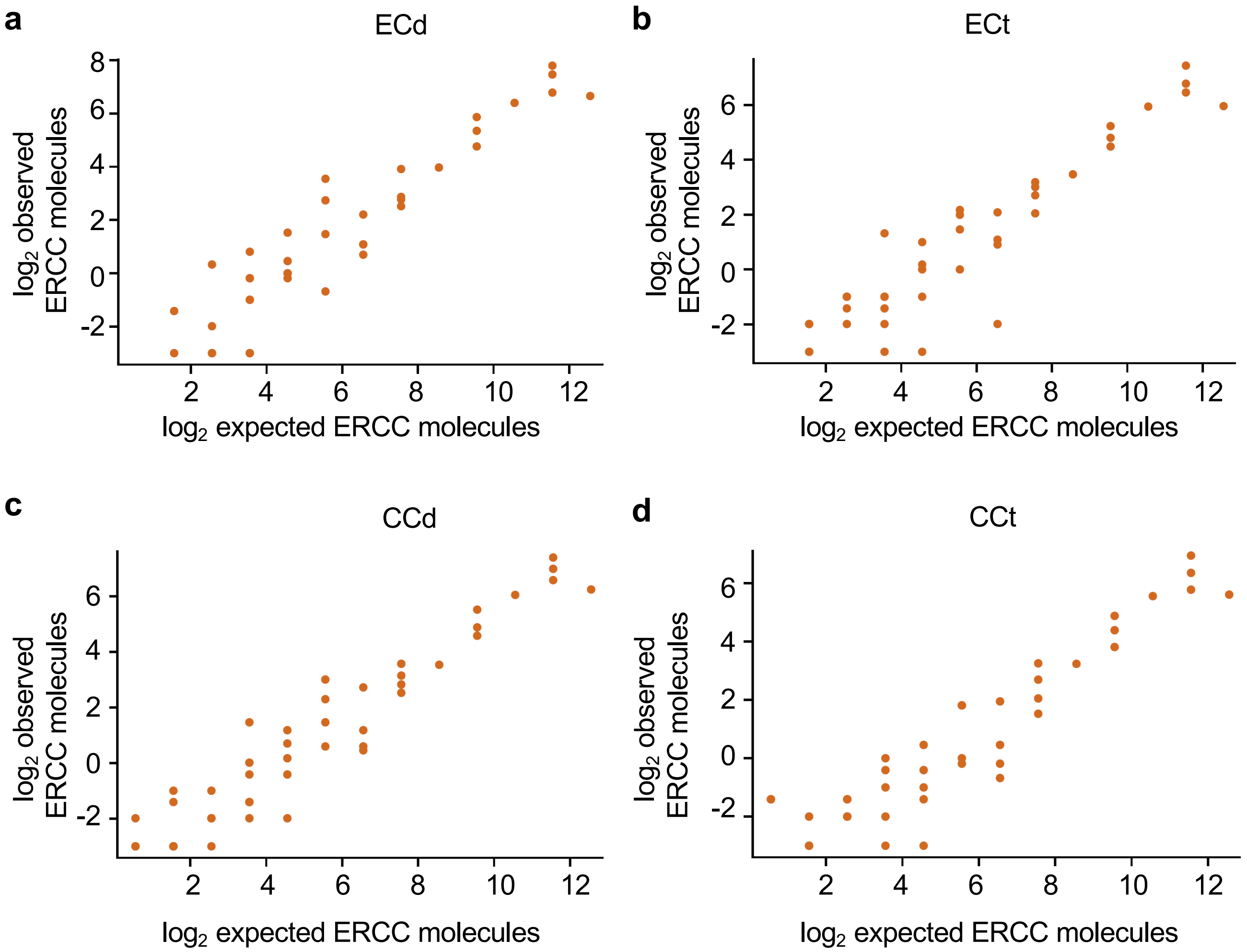

Additional file 1: Figure S5. Correlation of observed and expected molecule counts. **a-d** Observed molecule counts are plotted against the expected molecule counts for each ERCC RNA in ECd (**a**), ECt (**b**) CCd (**c**) and CCt (**d**).

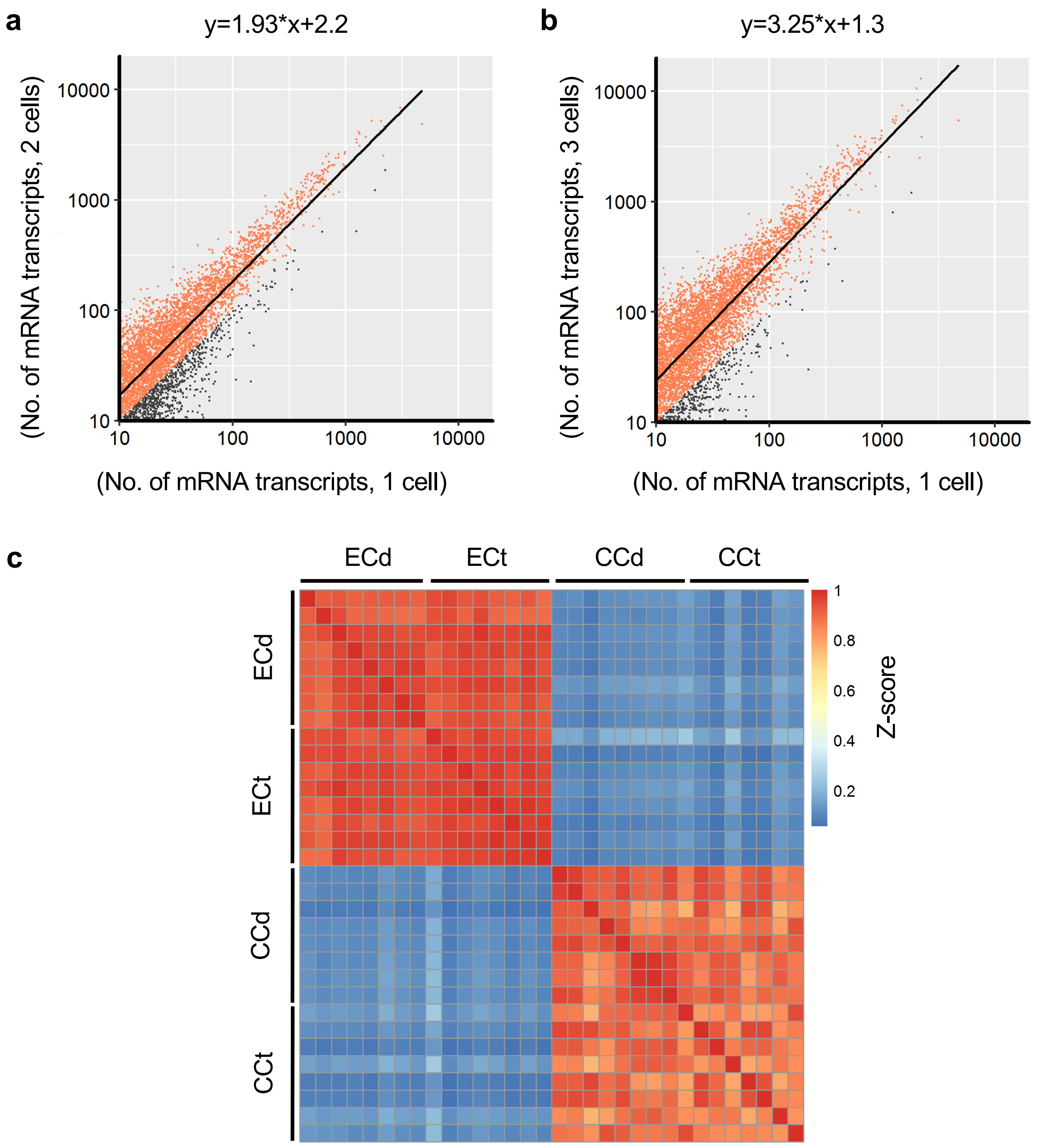

Additional file 1: Figure S6. Pairwise comparison of gene expression levels. **a,b** Pairwise comparison of normalized transcript number levels between one cell per library and two cells per library (**a**) and between one cell per library and three cells per library (**b**). Black lines indicate linear regression lines. Linear regression equations were displayed on the top of each panel. **c** Pairwise correlation coefficients of each cell of ECd, ECt, CCd and CCt which were calculated using absolute expression levels of all genes. ECd and ECt: egg cells in diploid and tetraploid plants, respectively; CCd and CCt: central cells in diploid and tetraploid plants, respectively.

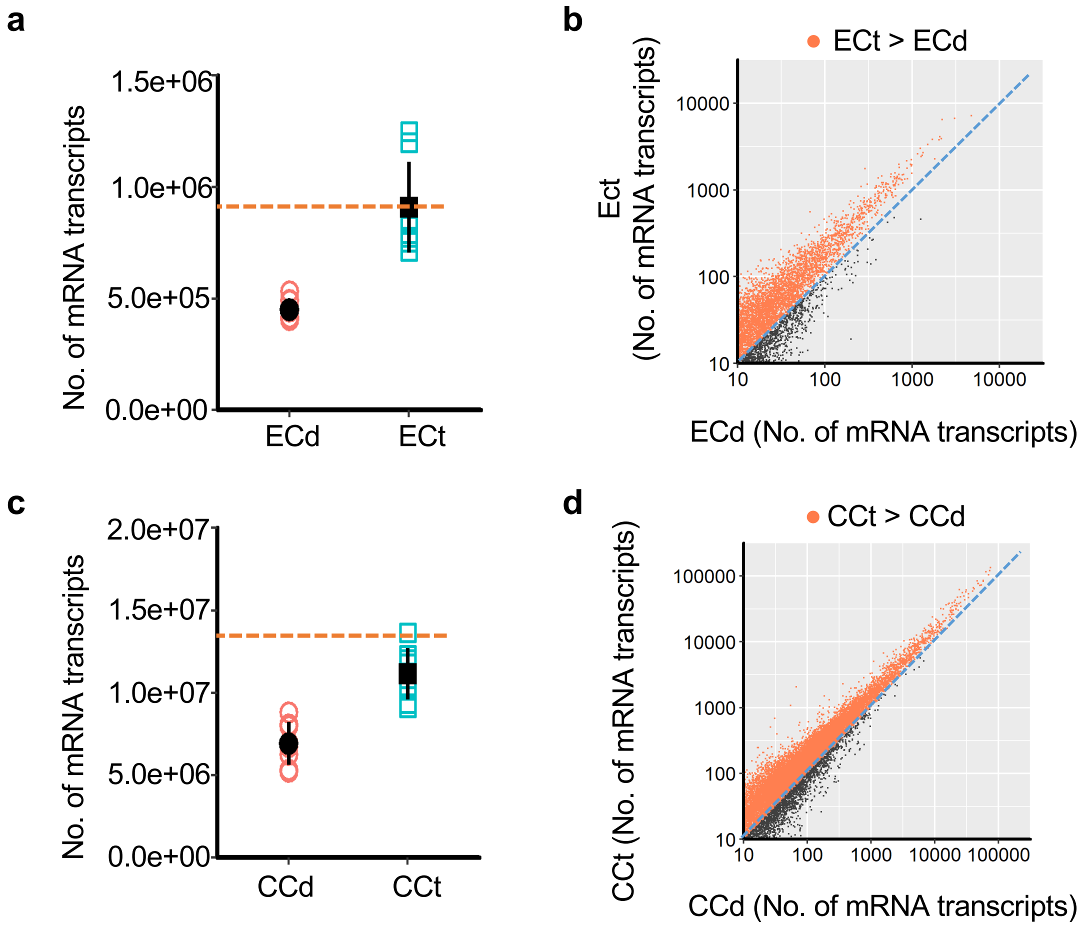

Additional file 1: Figure S7. Comparative analysis of transcripts in egg and central cells of diploid and tetraploid plants. **a, c** Total number of normalized mRNA transcripts in the ECd and ECt (**a**) and in the CCd and CCt (**c**). Black blocks indicate average mRNA amounts. Orange lines indicate 2-fold increase of transcripts in the tetraploid plant relative to the diploid plant. **b, d** Pairwise comparison of normalized transcript number levels in the egg cells of diploid and tetraploid plants (ECt vs ECd) (**b**) and in the central cells of diploid and tetraploid plants (CCt vs CCd) (**d**). Blue lines indicate no expression changes in each cell type between diploid and tetraploid plants. Orange dots indicate genes with numerically higher transcript levels. ECd and ECt: egg cells in diploid and tetraploid plants, respectively; CCd and CCt: central cells in diploid and tetraploid plants, respectively.

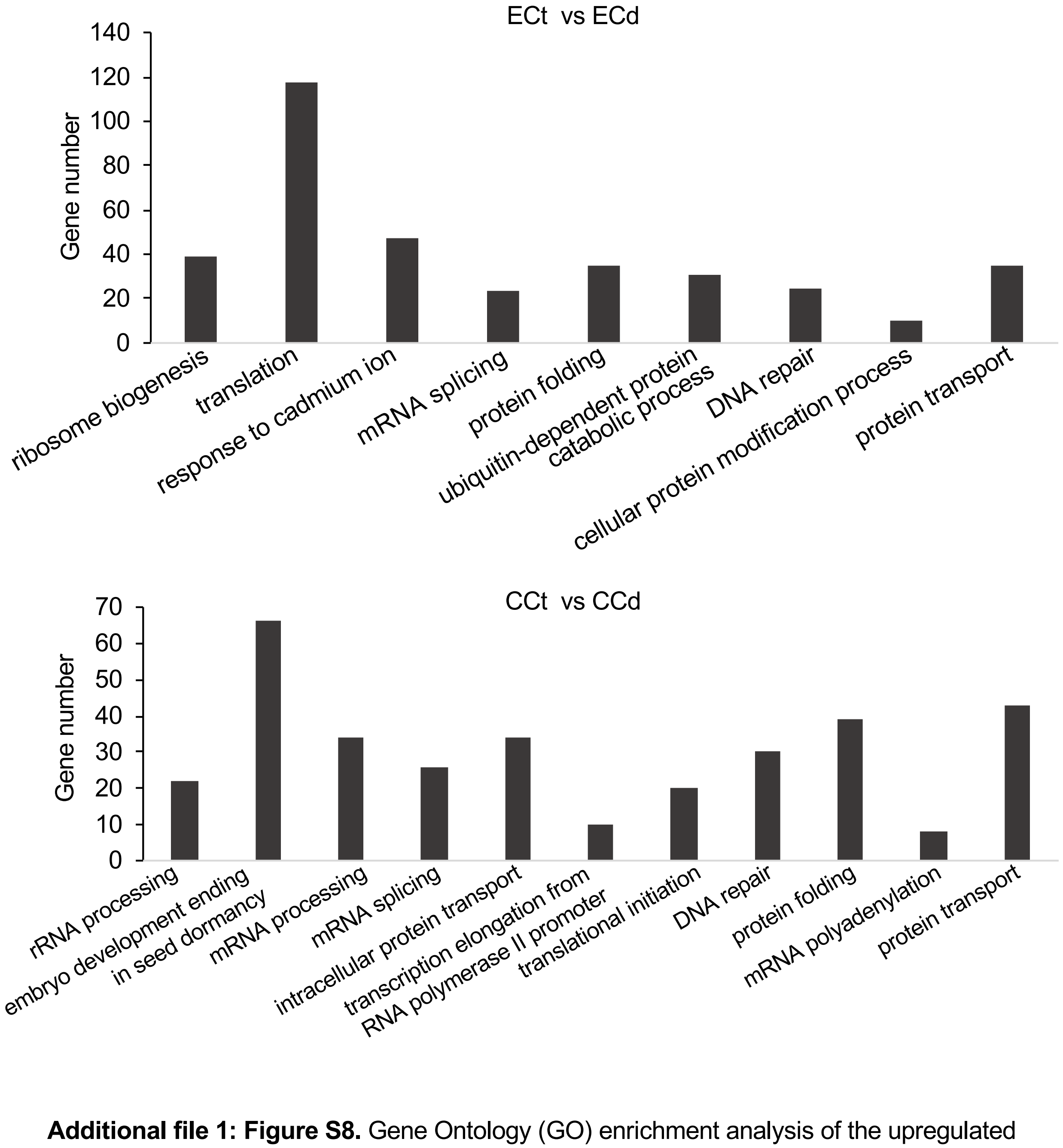

Additional file 1: Figure S8. Gene Ontology (GO) enrichment analysis of the upregulated genes in the egg cell (EC) or central cell (CC) of the diploid and tetraploid plants. ECd and ECt: egg cells in diploid and tetraploid plants, respectively; CCd and CCt: central cells in diploid and tetraploid plants, respectively.

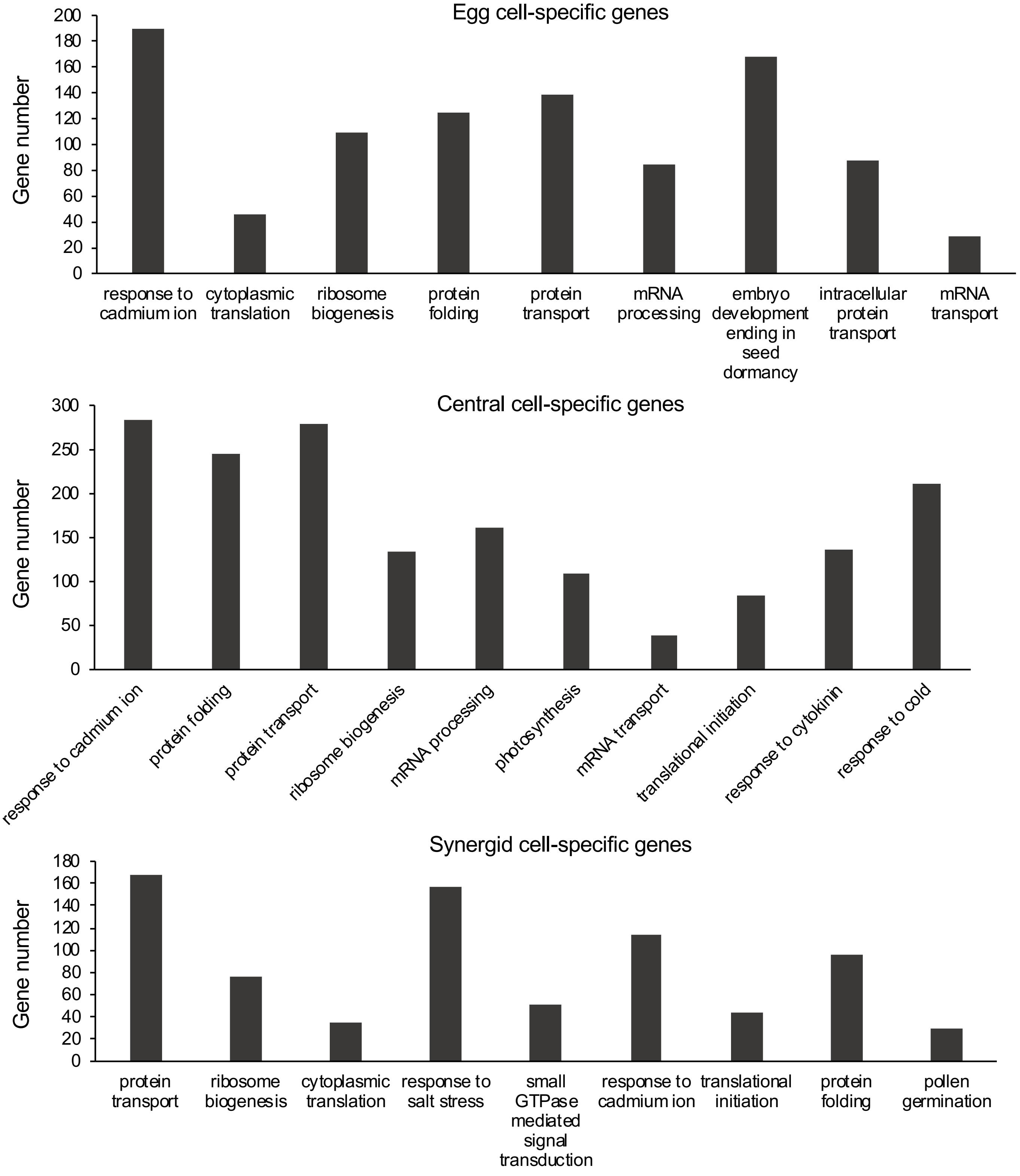

Additional file 1: Figure S9. Gene Ontology (GO) enrichment analysis of cell-type-specifically expressed genes in the egg, central, and synergid cells.
