# Supplementary material for: Single-cell RNA-seq analysis reveals ploidy-dependent and cell-specific transcriptome changes in Arabidopsis female gametophytes
Source: Genome Biol. 2020 Jul 22;21:178. doi: 10.1186/s13059-020-02094-0 (PMC7375004; doi:10.1186/s13059-020-02094-0)
Supplement: Supplementary file 6 — Additional file 6: Review history. [file 13059_2020_2094_MOESM6_ESM.docx]

Review History

**First round of review**

**Reviewer 1**

**Are you able to assess all statistics in the manuscript, including the appropriateness of statistical tests used?** Yes, and I have assessed the statistics in my report.

**Comments to author:**

This manuscript by Song et al. investigating gene expression changes due to polyploidy in gametic cells using single-cell RNA-seq is an appropriate and, as far as I know, a novel use of single cell transcriptomics applied to the field. I think the paper represents a nicely done introduction of this technique to the genome copy number field. The data is largely well verified and some interesting initial observations and confirmations come of out of the study. It is convincing that this is a better way to study the transcriptional effects of endoreplication than previous methods. I have a few comments on issues I think need some attention.

1. Number of cells. As far as I could tell, one has to dig into the supplemental tables to determine exactly how many cells of each cell type were sampled. It appears to be 8 in most cases and four in one case. Its a small sample size but the authors use more high depth single-cell profiling techniques. So, that number should be stated in early results. It would help to do some kind of resampling Monte Carlo test to ensure that this number of samples has a small enough confidence interval on critical analytical results to ensure sample size is not an issue. e.g., fold changes in specific cell types due to endoreplication.

2. The presentation of transcript numbers gets a bit confusing. On line 153, UMI counts are listed at 25,000 per cell. On line 218, the ms states the central cell has almost 7 million transcripts. Below other cell types are also stated as having transcript abundances in the millions. I know there is an extrapolation based on spike ins and capture rate (those methods seem fine), but the authors should be careful about stating exactly what the information and derived figures they are presenting at any given section. It could get confusing. I then have some trouble connecting the data in the text with the graph in fig. 3a, which appears to top off at 1.3 million?

3. The authors use several statistical approaches that appear appropriate to me, but materials and methods lacks detail on how those were done. e.g., ANOVA, that must be a version that inputs count data, e.g, deseq or some similar program. State the programs used or how it was done otherwise.

I don't think more experiments need to be done on any of the biological results. This is a methodological paper that demonstrates the use of technique applied to an important field. The intriguing biological results can be pursued in follow ups. I think the main issue is presenting a complete validation of the technique, which can be done with clarification and perhaps a little more analysis, as per comment 1.

**Reviewer 2**

**Are you able to assess all statistics in the manuscript, including the appropriateness of statistical tests used?** Yes, and I have assessed the statistics in my report.

**Comments to author:**

In the manuscript „Single-cell RNA-seq analysis reveals ploidy dependent and cell-specific transcriptome changes in the Arabidopsis female gametophytes" the authors present a transcriptional investigation of individual cells of the mature gametophyte (egg cell, central cell, synergids) of Arabidopsis thaliana and compared transcript numbers at diploid and tetraploid levels for egg and central cells. The study further describes correlations between transcript abundance, cell size and ploidy and an increased activity of DEMETER in the tetraploid central cell as compared to the diploid, resulting in derepression of Polycomb-Group genes, which is affecting target gene expression. In addition, abundant transcription of genes encoding for cystein-rich peptides is reported.

The subject of the study is of interest, especially from the perspectives of plant reproduction, polyploidization and evolution. Also the technical approach of single cell RNA-Seq to evaluate transcript abundance is state of the art. However, I have certain concerns regarding aspects of the presentation of data, methods, and conclusions as outlined below:

(I) Importantly, presentation of the study design and methods is not consistently sufficient. More details on the bioinformatic data analysis should be given, starting with the tools used for quality control and trimming of the raw reads, normalization and ANOVA, principal component analysis, and for the analysis of the published datasets from laser microdissection (i.e. if the raw reads have been taken and mapped to the same reference as the data from this study), and for the gene ontology (GO) analysis. Concerning the GO analysis details on statistics would much improve the presentation.

Also, it would be greatly helpful if details would be given if the biological replicates have been collected from the same or different plants. Information should be provided on how many biological replicates have been collected for the samples comrising 2 or 3 cells per library.

(II) Even though the study design of the single cell RNA-Seq with the use of UMI and ERCC spike in controls is generally convincing, an even more careful evaluation of data quality would be benificial. This is in particular the case, as due to the large differences in transcript abundance observed between cell types, a technical effect on estimation of transcript levels during normalization cannot fully be excluded. Also, it is not obvious if the full set of ERCC spike in transcripts has been used for normalization, making the evaluation of the procedure difficult. As the identification of low expressed genes is in general a technical challenge in RNA-Seq with very small amounts of RNA input, rather than the comparison of total numbers of RNA transcripts (Figure 3A), also the presentation of correlation of gene expression normalized by the number of cells used in the samples might provide additional useful information. This also relates to lines 198ff: Here it would be interesting to evaluate if the effects of higher expression are related to the expression levels or more or less equally distributed.

As only 4 genes per egg cell and central cell were taken into account in the analysis presented in Figure S4 and line 136ff, the support of a „good representation of cell specific transcriptomes" could be much improved by taking more genes into account that have been previously reported and validated to be active in a certain cell type of the female gametophyte (i.e.  Steffen et al, Plant J. 2007 and others).

Also, a broader comparison of cell type specific transcriptomes of diploid cells from this study to previously reported transcriptome profiles obtained by laser assisted microdissection and RNA-Seq might be helpful to evaluate the single cell RNA-Seq presented.

(III) Conclusions drawn from the data appear not always sufficiently supported. This could be improved by more careful description but also by additional data analysis.

This concerns in particular the presentation of correlation of cell size, ploidy level and transcript abundance and the presentation of OSR2 and TOR as putative regulators. As the increase in expression of OSR2 and TOR in tetraploid cells as compared to diploid follows the global trend observed for the majority of genes, from the data presented in this study no clear evidence is provided for their involvement in cell size regulation. The discussion of evidence from the literature would be better suited to the discussion section (lines 199-211).

While the differences presented between number of transcripts per cell are impressive in comparison of the diploid egg and central cell, estimates on a correlation of cell size and transcript abundance might be improved by also taking the diploid synergids into account of which transcriptome data have been obtained (lines 216ff).
The manuscript would benefit from providing more details on how the abundant transcription of genes encoding cystein rich proteins has been determined, but also from a more detailed discussion of previously reported evidence of the abundance and roles of the genes in female gametophytes. In particular with respect to this aspect, the advance of the data presented over previous studies would become clearer.

Minor Comments:
-     Although I do not doubt the differences presented in Figures 4c, d, I am missing a statistical test here.
-     Figure 5: If it is TPM normalized mean expression levels shown here this should be indicated.
-     Lines 243ff: Some statistical test of differential expression would be meaningful here. It remains unclear how the top 20 genes uniquely expressed were determined. This should be described clearly.
-     Line 246: Figure 5A?
-     The information provided in line 244 („in any library") and legend Figure 5 („more than half of the examined cells") is not consistent.
-     Revising the manuscript to remove certain redundancies in information, e.g. lines 313ff and 187ff would be beneficial.
-     The nomenclature of „female gamete specifically expressed genes" can be mis-leading, as the study does not include any comparisons to other datasets from additional tissues. Therefore these genes are genes only detected in either egg cell, central cell, or synergids, but not necessarily specific to these cells.

**Authors Response**

**Point-by-Point Response (in boldface letters)**

Reviewer #1: This manuscript by Song et al. investigating gene expression changes due to polyploidy in gametic cells using single-cell RNA-seq is an appropriate and, as far as I know, a novel use of single cell transcriptomics applied to the field. I think the paper represents a nicely done introduction of this technique to the genome copy number field. The data is largely well verified and some interesting initial observations and confirmations come of out of the study. It is convincing that this is a better way to study the transcriptional effects of endoreplication than previous methods. I have a few comments on issues I think need some attention.

**Response: We appreciate the positive and supportive assessment of our work by this expert reviewer.**

1. Number of cells. As far as I could tell, one has to dig into the supplemental tables to determine exactly how many cells of each cell type were sampled. It appears to be 8 in most cases and four in one case. Its a small sample size but the authors use more high depth single-cell profiling techniques. So, that number should be stated in early results. It would help to do some kind of resampling Monte Carlo test to ensure that this number of samples has a small enough confidence interval on critical analytical results to ensure sample size is not an issue. e.g., fold changes in specific cell types due to endoreplication.

**Response: To clarify this, we included the information of cell numbers in early part of the results (Page 6, line 123). “For egg cell and central cell in diploid and tetraploid plants, scRNA-seq libraries were constructed using eight cells (biological replicates) for each cell type as previously described [25, 35] (Methods). In addition, four synergid cells in the diploid plants were used for scRNA-seq library construction and analysis.” In addition, we clarified the replicates in artificial mix of egg cells. “To confirm the reproducibility, we made additional RNA-seq libraries using an artificial mix of two or three egg cells in each library, each with five biological replicates.”**

**Thanks for this suggestion. Using the Monte Carlo Simulation, we confirmed the significant expression changes by genome duplication**. **“For egg cells, normalized mRNA transcripts per cell were ~449,836 in the diploid** **plant (Additional file 1: Fig. S7a, b), which were doubled (~909,969) in the tetraploid plant (*P* = 0.0001, Monte Carlo simulation, *N* = 10,000), indicating that genome duplication has a doubling effect on the transcriptome abundance in the egg cell.” This is immediately followed by**, **“However, the central cell of the tetraploid plant showed ~1.6-fold increase of the transcript abundance (11,156,304) compared with that of the diploid plant (*P* = 0.0002, Monte Carlo simulation, *N* = 10,000)”.**

2. The presentation of transcript numbers gets a bit confusing. On line 153, UMI counts are listed at 25,000 per cell. On line 218, the ms states the central cell has almost 7 million transcripts. Below other cell types are also stated as having transcript abundances in the millions. I know there is an extrapolation based on spike ins and capture rate (those methods seem fine), but the authors should be careful about stating exactly what the information and derived figures they are presenting at any given section. It could get confusing. I then have some trouble connecting the data in the text with the graph in fig. 3a, which appears to top off at 1.3 million?

**Response: This is a good comment. We clarified this and moved it upfront in the revision. “By counting raw number of observed UMIs, we detected ~25,000 transcripts per egg cell and ~230,000 transcripts per central cell (Additional file 2: Table S1). Based on the abundance of all ERCC spike-in controls, we found that the capture efficiency of mRNA in scRNA-seq libraries varied from ~2% to ~7%. To eliminate the effect of capture efficiency variation and more importantly to compare expression abundance among different cell types, we normalized average transcript abundance of each gene per egg or central cell using capture efficiency (Methods). …. For example, the above raw numbers were normalized as average transcript abundance of 449,836 per egg cell and 6,924,840 per central cell. These normalized values were used for further analysis.”**

**After this description, it should be easy to follow the text and data presentation in Fig. 3a. The purpose of Fig. 3a is to show that scRNA-seq data are reproducible by technical replications by artificially mixing 2 and 3 cells in each sample, each with 5 biological replicates. As shown in Fig. 3a, the normalized transcript abundance was in a good linear relationship from 1 egg cell (~449,836 ) to 3 egg cells (~1,507,362). For clarity, we also added “Number of cells per library” in the *X*-axis of Fig. 3a.‬‬‬**

3. The authors use several statistical approaches that appear appropriate to me, but materials and methods lacks detail on how those were done. e.g., ANOVA, that must be a version that inputs count data, e.g, deseq or some similar program. State the programs used or how it was done otherwise.

**Response: In the Methods, we added a short paragraph, entitled “Identification of upregulated genes by genome doubling,” with the following description, “Data in each cell was treated as a biological replicate. Normalized expression abundance of each gene with eight replicates was subjected to one-way ANOVA test using f_oneway function in SciPy software (**[**https://www.scipy.org**](https://www.scipy.org)**) to identify upregulated genes between ECt and ECd or between CCt and CCd with a statistical significance level of** ***P* < 0.05.” We also updated PCA, GO analysis, and normalization methods, as also suggested by Reviewer 2.**

I don't think more experiments need to be done on any of the biological results. This is a methodological paper that demonstrates the use of technique applied to an important field. The intriguing biological results can be pursued in follow ups. I think the main issue is presenting a complete validation of the technique, which can be done with clarification and perhaps a little more analysis, as per comment 1.

**Response: Many thanks for this informative and positive summary by the expert reviewer. We believe that all comments have been addressed as shown above.**

Reviewer #2: In the manuscript „Single-cell RNA-seq analysis reveals ploidy dependent and cell-specific transcriptome changes in the Arabidopsis female gametophytes" the authors present a transcriptional investigation of individual cells of the mature gametophyte (egg cell, central cell, synergids) of Arabidopsis thaliana and compared transcript numbers at diploid and tetraploid levels for egg and central cells. The study further describes correlations between transcript abundance, cell size and ploidy and an increased activity of DEMETER in the tetraploid central cell as compared to the diploid, resulting in derepression of Polycomb-Group genes, which is affecting target gene expression. In addition, abundant transcription of genes encoding for cystein-rich peptides is reported.

The subject of the study is of interest, especially from the perspectives of plant reproduction, polyploidization and evolution. Also the technical approach of single cell RNA-Seq to evaluate transcript abundance is state of the art. However, I have certain concerns regarding aspects of the presentation of data, methods, and conclusions as outlined below:

**Response: We appreciate the positive and constructive assessment of our work by this expert reviewer and have addressed the concerns as follows.**

(I) Importantly, presentation of the study design and methods is not consistently sufficient. More details on the bioinformatic data analysis should be given, starting with the tools used for quality control and trimming of the raw reads, normalization and ANOVA, principal component analysis, and for the analysis of the published datasets from laser microdissection (i.e. if the raw reads have been taken and mapped to the same reference as the data from this study), and for the gene ontology (GO) analysis. Concerning the GO analysis details on statistics would much improve the presentation.

**Response: We amended details of the methods for all analytical procedures, including ANOVA, PCA, analysis of published LCM datasets and GO terms in the Methods.**

**Identification of up-regulated genes by genome doubling**

**Data in each cell were treated as a biological replicate. Normalized expression abundance of each gene with eight replicates was subjected to one-way ANOVA test using f_oneway function in SciPy software (**[**https://www.scipy.org**](https://www.scipy.org)**) to identify upregulated genes between ECt and ECd or between CCt and CCd with a statistical significant level of P < 0.05.**

**Principal Component Analysis (PCA) and Gene Ontology (GO) analysis**

**Normalized expression abundance of genes in egg cells and central cells from diploid and tetraploid plants was subject to logarithm transformation using *log2* function in R software. PCA was performed using *prcomp* function in R software with the parameters “scale. = FALSE, center = FALSE, tol = 0”.**

**GO analysis was performed using The Database for Annotation, Visualization and Integrated Discovery (DAVID) v6.8 (‘**[**https://david.ncifcrf.gov/home.jsp**](https://david.ncifcrf.gov/home.jsp)**’) {Huang da, 2009 #11540}. The enriched biological process terms were extracted from GOTERM_BP_DIRECT category with Benjamini-corrected P value < 0.05.**

**Analysis of published LCM datasets and genes expressed in female gametes**

**Raw reads from LCM datasets were mapped to *Arabidopsis* genome (TAIR 10) using HISAT2 (v2.1.0) software with default parameters {Kim, 2019 #11538}. Uniquely mapped reads were extracted and used to calculate transcripts levels (Transcripts Per Million, TPM) of each gene through StringTie (v1.3.3) software {Pertea, 2015 #11539}.**

**To compare scRNA-seq datasets with LCM datasets, we calculated the TPM value of each gene in scRNA-seq datasets. For each gene, total number of distinct UMIs was calculated as transcript number. The sum of transcript numbers of all genes was divided by 1,000,000 to create a scaling factor. Transcript number of each gene is divided by the scaling factor to generate the TPM value of each gene.”**

Also, it would be greatly helpful if details would be given if the biological replicates have been collected from the same or different plants. Information should be provided on how many biological replicates have been collected for the samples comrising 2 or 3 cells per library.

**Response: Single cells were collected from different plants in various times. Five biological replicates were used for each of the samples containing 2 or 3 cells per library. There are 8 biological replicates for 1 cell library and all other libraries, except for synergid cells with 4 biological replicates. We have clarified those in the Methods and Results.**

(II) Even though the study design of the single cell RNA-Seq with the use of UMI and ERCC spike in controls is generally convincing, an even more careful evaluation of data quality would be benificial. This is in particular the case, as due to the large differences in transcript abundance observed between cell types, a technical effect on estimation of transcript levels during normalization cannot fully be excluded. Also, it is not obvious if the full set of ERCC spike in transcripts has been used for normalization, making the evaluation of the procedure difficult. As the identification of low expressed genes is in general a technical challenge in RNA-Seq with very small amounts of RNA input, rather than the comparison of total numbers of RNA transcripts (Figure 3A), also the presentation of correlation of gene expression normalized by the number of cells used in the samples might provide additional useful information. This also relates to lines 198ff: Here it would be interesting to evaluate if the effects of higher expression are related to the expression levels or more or less equally distributed.

**Response: We used the full set of ERCC spike-in transcripts for normalization. We clarified this by adding additional details of the normalization method in the Methods.**

**“The slope (**$\boldsymbol{\beta}$**_1_) and intercept (**$\boldsymbol{\beta}$**_0_) of ERCC spike-in controls in each library were calculated using *glm* function in R software as “glm(y~log2(x), family=’poisson’)” in which ‘x’ and ‘y’ were *Mol_exp_* and *Mol_obs_* , respectively, of the** **of ERCC spike in transcripts.”**

**Indeed, it is a challenge to accurately detect expression levels of poorly expressed genes in scRNA-seq. We made a new figure (Fig. S6a, b) to explain this and moved previous Fig. S4b to Fig. S6c for sequential order of citation in the text. Contextually these pieces fit in one figure. We added the following description in the revision.** **“The correlation between expression levels and cell numbers were further analyzed by pairwise comparison of mRNA transcript number levels among libraries containing different numbers of cells** **(Additional file 1: Fig. S6a, b)**. **Linear regression analysis showed two and three cells per library had 1.93-fold and 3.25-fold increase of transcript numbers, respectively, compared with one cell per library; highly expressed genes showed better correlation between transcript abundance and cell numbers than poorly expressed genes.”**

**As suggested, we added one sentence with respect to line 198, Fig. S7b,d (Previous Fig. S6b,d). “At the genome-wide level, 57% and 58% of the expressed genes (>3,000) showed larger than 1.5-fold increase after genome duplication in egg and central cells, respectively (Additional file 2: Table S1). The genes with higher expression levels tended to show more expression increase than the genes with lower expression levels after genome doubling.”**

As only 4 genes per egg cell and central cell were taken into account in the analysis presented in Figure S4 and line 136ff, the support of a „good representation of cell specific transcriptomes" could be much improved by taking more genes into account that have been previously reported and validated to be active in a certain cell type of the female gametophyte (i.e. Steffen et al, Plant J. 2007 and others).

**Response: As suggested, we added more cell-specific genes (6 in egg cell and 12 in central cell) using the data from Steffen et al. (2007, Plant J) and Sprunck et al. (2012, Science) and re-plot Fig. S4a using normalized transcript abundance in the revision.**

Also, a broader comparison of cell type specific transcriptomes of diploid cells from this study to previously reported transcriptome profiles obtained by laser assisted microdissection and RNA-Seq might be helpful to evaluate the single cell RNA-Seq presented.

**Response: As suggested, we added ten more cell-specific genes and re-plot Fig. 5a using TPM values. The gene IDs were updated in the revised text.**

(III) Conclusions drawn from the data appear not always sufficiently supported. This could be improved by more careful description but also by additional data analysis.

This concerns in particular the presentation of correlation of cell size, ploidy level and transcript abundance and the presentation of OSR2 and TOR as putative regulators. As the increase in expression of OSR2 and TOR in tetraploid cells as compared to diploid follows the global trend observed for the majority of genes, from the data presented in this study no clear evidence is provided for their involvement in cell size regulation. The discussion of evidence from the literature would be better suited to the discussion section (lines 199-211).

**Response: As suggested, in the revision, we moved the literature discussion part of the cell size regulation to the middle of the second paragraph in the Discussion.**

While the differences presented between number of transcripts per cell are impressive in comparison of the diploid egg and central cell, estimates on a correlation of cell size and transcript abundance might be improved by also taking the diploid synergids into account of which transcriptome data have been obtained (lines 216ff).

**Response: This is a good suggestion. We added the following information in the revision.** **“In addition, the synergid cell in the diploid plant had ~66,773 raw transcripts or** **~2.9 million normalized mRNA transcripts. This suggests that the synergid cell contains more transcripts than the egg cell but less than the central cell, which is consistent with the published observation that the synergid cell is larger than the egg cell but smaller than the central cell {Englhart, 2017 #11541}. These results suggest a positive correlation between RNA content and cell size [57].”**

The manuscript would benefit from providing more details on how the abundant transcription of genes encoding cystein rich proteins has been determined, but also from a more detailed discussion of previously reported evidence of the abundance and roles of the genes in female gametophytes. In particular with respect to this aspect, the advance of the data presented over previous studies would become clearer.

**Response: We added TPM calculation of each gene from scRNA-seq in the Methods. “To compare our scRNA-seq datasets with the LCM datasets, we calculated the TPM value of each gene in the scRNA-seq datasets. For each gene, total number of distinct UMIs was calculated as transcript number. The sum of transcript numbers of all genes was divided by 1,000,000 to create a scaling factor. Transcript number of each gene is divided by the scaling factor to generate the TPM value of each gene.”**

**With respect to CRPs, we updated the relevant information in the Discussion. “Compared to previous studies, our scRNA-seq datasets are largely free of cross-contamination among three female gametophytic cells, which provide a unique resource for plant reproductive biology. … CRPs are involved in diverse aspects of cell-cell communication during vegetative growth and plant reproduction {Marshall, 2011 #11188}{Dresselhaus, 2013 #11150}. As the amino acid composition of CRPs is highly divergent, different CRPs may play specific and unique functions in the egg cell, central cell, or synergid cell during fertilization. For example, CRPs secreted from synergid cells can provide guidance for pollen tube and sperm cell release, while those from the central cell and egg cells can attract sperm to produce embryo and endosperm of a seed {Tian, 2005 #11262}{Dresselhaus, 2013 #11150}, a process known as double fertilization {Brink, 1940 #4692}{Berger, 2008 #11261}. These newly identified female gamete-expressed CRPs are key signaling factors in regulating reproductive development in plants. Compared to the egg cell, the function of CRPs in the central cell and synergid cell is poorly understood. Our scRNA-seq resource should provide valuable guidelines for future research directions to elucidate the roles of egg, central, and synergid cells during double fertilization [58, 68].”**

Minor Comments:

- Although I do not doubt the differences presented in Figures 4c, d, I am missing a statistical test here.

**Response: Student’s *t* test was used to confirm significant upregulation of expression levels of *OSR2* and *TOR* by genome doubling (*P* < 0.01), which was added to Fig 4c, d.**

- Figure 5: If it is TPM normalized mean expression levels shown here this should be indicated.

**Response: We added description in figure legend, “Gene expression levels in scRNA-seq were mean transcript per million (TPM) values of the genes in all cells for each cell type.”**

- Lines 243ff: Some statistical test of differential expression would be meaningful here. It remains unclear how the top 20 genes uniquely expressed were determined. This should be described clearly.

**Response: As suggested, we used ANOVA for the statistical test. “A total of 12,738 genes (39% of all expressed genes) showed significantly different expression patterns in at least two of the three (egg, central, and synergid) cell types (*P* < 0.05, one-way ANOVA), indicating enormous expression variation among female gametes”.**

**Top 20 genes refer to “top 20 genes with the highest expression values.”**

- Line 246: Figure 5A?

**Response: Thanks. Fig 3*A* was a typo, which should be Fig. 5a.**

- The information provided in line 244 („in any library") and legend Figure 5 („more than half of the examined cells") is not consistent.

**Response: Sorry for the mistake. We revised the legend as “An expressed gene is defined as its expression in one or more cells examined.”**

- Revising the manuscript to remove certain redundancies in information, e.g. lines 313ff and 187ff would be beneficial.

**Response: As suggested, we removed the sentence about *dme* from the Results and now in the Discussion. We also moved cell size regulation part in the Results to Discussion.**

- The nomenclature of „female gamete specifically expressed genes" can be mis-leading, as the study does not include any comparisons to other datasets from additional tissues. Therefore these genes are genes only detected in either egg cell, central cell, or synergids, but not necessarily specific to these cells.

**Response: As suggested, we removed “specifically” and revised to “female gamete-expressed genes” for the genes identified in scRNA-seq analysis.**

**Second round of review**

**Reviewer 1**

My main concern on statistical rigor was addressed with the monte carlo simulation that upheld the original conclusions. I took a look at all reviewer comments and I think the authors have addressed the important issues raised, which mainly deal with clarity and providing more detail on the results. My issues have been addressed and I have no remaining concerns.

**Reviewer 2**

The revised manuscript „Single-cell RNA-seq analysis reveals ploidy-dependent and cell-specific transcriptome changes in Arabidopsis female gametophytes“ presented by the authors represents an improved version of the manuscript. I have no further comments.
